# Supplementary material for: Characterization of a methyltransferase for iterative N-methylation at the leucinostatin termini in Purpureocillium lilacinum
Source: Commun Biol. 2024 Jun 22;7:757. doi: 10.1038/s42003-024-06467-0 (PMC11193748; doi:10.1038/s42003-024-06467-0)
Supplement: Supplementary file 2 — Supplementary Information [file 42003_2024_6467_MOESM2_ESM.pdf]

Characterization of a methyltransferase for iterative *N*-methylation at the leucinostatin termini in *Purpureocillium lilacinum*

Zixin Li[1,2], Yang Jiao[1], Jian Ling[1], Jianlong Zhao[1], Yuhong Yang[1], Zhenchuan Mao[1], Kaixiang Zhou[3], Wenzhao Wang[4], Bingyan Xie[1,*], Yan Li[1,*]

[1]State Key Laboratory of Vegetable Biobreeding, Institute of Vegetables and Flowers, Chinese Academy of Agricultural Sciences, Beijing, 100081, China

[2]Microbial Processes and Interactions (MiPI), TERRA Teaching and Research Centre, Gembloux Agro-Bio Tech, University of Liège, Gembloux, 5030, Belgium

[3]Center for Advanced Materials Research, Advanced Institute of Natural Sciences, Beijing Normal University at Zhuhai, Zhuhai, 519087, China

[4]State Key Laboratory of Mycology, Institute of Microbiology, Chinese Academy of Sciences, Beijing, 100101, China

These authors contributed equally: Zixin Li, Yang Jiao

* Corresponding authors: B. Xie; Y. Li.

Email: xiebingyan@caas.cn; liyan05@caas.cn.

Contents

Supplementary Tables

Supplementary Table 1. Comparison of the reported fragment ions (*m/z)* in ESI-MS-MS spectra of leucinostatins in this study. **3**

Supplementary Table 2. The pLDDT scores of six predicted active residues. **4**

Supplementary Table 3. RMSD values of LcsG and its homologies. **5**

Supplementary Table 4. The system setup that includes number of simulations per system, simulation box dimensions, total number of atoms, total number of water molecules, salt concentration, liquid composition (number of molecules and type). **6**

Supplementary Table 5. Known NMTs capable of furnishing at least two methyltransfers to at least one substrate and their similarity to LcsG. **7**

Supplementary Table 6. Annotated enzymes clustered with LcsG in the sequence similarity network. **8**

Supplementary Table 7. Non-ribosomal peptide synthetases (NRPSs) contained terminal modules similar to that of LcsG. **10**

Supplementary Table 8. Plasmids and strains used in this study. **11**

Supplementary Table 9. Primers used in this study. **12**

Supplementary Figures

Supplementary Figure 1. The scheme of plasmids construction. **13**

Supplementary Figure 2. Confirmation of the lcsG deletions in *P. lilacinum* strain PLBJ-1. ....**14**

Supplementary Figure 3. Quantitative PCR and LC-MS analysis of *lcsG* overexpression mutants. **15**

Supplementary Figure 4. HRESI-MS spectrum of LeuC (**1**), LeuB (**2**), LeuA (**3**), and LeuK0 (**4**). **16**

Supplementary Figure 5. Fragmentation types observed in MS-MS spectra of leucinostatins. **17**

Supplementary Figure 6. ^{13}C-NMR (APT) of LeuK0 in CD_3OD. **18**

Supplementary Figure 7. ^{13}C-NMR (DEPT-135) of LeuK0 in CD_3OD. **19**

Supplementary Figure 8. ^{13}C-NMR (DEPT-90) of LeuK0 in CD_3OD. **20**

Supplementary Figure 9. NHS ester reaction scheme. **21**

Supplementary Figure 10. LC-MS detection of SAM and SAH of LcsG-catalyzed reactions. **22**

Supplementary Figure 11. $[M+H]^+$ ions of LeuK2 (**6**), LeuK3 (**7**) and LeuA0 (**8**). **23**

Supplementary Figure 12. Effect of pH (a) and temperature (b) on the production of LeuA0 (**8**) and LeuK3 (**7**) by LcsG- catalyzed. ..**24**

Supplementary Figure 13. Standard curve of concentration and peak area of LeuA0 (**8**) and LeuK3 (**7**) determined by LC-MS............**25**

Supplementary Figure 14. Ions from the HRESI-MS-MS data (a) and predicted fragments (b) of LeuB (**2**), LeuA (**3**). **26**

Supplementary Figure 15. Ions from the HRESI-MS-MS data of LeuK0 (**4**), LeuK2 (**6**), and LeuK3 (**7**). **27**

Supplementary Figure 16. MIC (μg/mL) of leucinostatins (LeuA (**3**), LeuA0 (**8**), LeuK0 (**4**), LeuK3 (**7**)) against *Cryptococcus neoformans* strain H99 in a 2-fold liquid dilution assay. **28**

Supplementary Figure 17. Comparison between the LcsG and its structurally characterized homologues. **29**

Supplementary Figure 18. Structural analysis of LcsG: pLDDT scores (a), dimer configuration (b), and active site residues (c) **31**

Supplementary Figure 19. Formulas and sequences of LeuA, ZHAWOC6027, and helioferin A. **32**

Supplementary Figure 20. The results of Diffdock analysis utilizing the modified LeuA structure and SAH. **33**

Supplementary Figure 21. 500 ns trajectories protein & ligand RMSD. Protein and ligand were stable after 500 ns MD simulation. **34**

Supplementary Figure 22. Interatomic distance between targeted N of LeuA (LeuA:68) and two O of D368 (OD1 and OD2) in 3 independent MD simulations. **35**

Supplementary Figure 23. Representative snapshot from molecular dynamics simulations depicting the formation of salt bridges between the ligand and D368 and D395. ....**36**

Supplementary Figure 24. The DNA sequencing of the plasmids (a) and western blot analysis (b) of LcsG mutagenesis. **37**

Supplementary Figure 25. Sequence comparison between the LcsG and other identified NMTs and OMTs. **38**

Supplementary Figure 26. (a) A phylogenetic tree showing the relationships between LcsG and other methyltransferases. (b) Alignment sequences of OMT (P16559) and NMT (RosA). **39**

Supplementary Figure 27. (a) Sequence similarity network (SSN) of LcsG and other fungi-derived methyltransferases from PF00891. (b) Substrates of the NMT FsaA, PynC, EqxD, and Phm5. ....**41**

Supplementary Figure 28. The termination step mediated by the reductase (R) domain in the biosynthesis of NRPs whose gene contained similar terminal modules with that of LcsG. **42**

Supplementary Figure 29. Uncropped images of Fig. 2a, Supplementary Fig. 2, and Supplementary Fig. 24b. **43**

Supplementary Tables

Supplementary Table 1. Comparison of the reported fragment ions (m/z) in ESI-MS-MS spectra of leucinostatins in this study.

	**B1**	**A2**	**B2**	**B3**	**B4**	**B5**	**B6**	**B7**	**B8**	**B9**	**C10**	**Y5**	**Y4**	**Y3**	**Y2**
LeuA	111	194	222	435	564	649	762	875	960	1045	1173	457	344	259	174
LeuB	111	194	222	435	564	649	762	875	960	1045	1173	443	330	245	160
LeuC	111	194	222	435	564	649	762	875	960	1045	1173				
LeuA0	111	194	222	435	564	649	762	875	960	1045	1173				
LeuK0	111	194	222	435	564	649	762	875	960	1045	1173	473	360	275	190
Unknown compound	111	194	222	417	546	613	744	857	942	1027	1155	473	360	275	190
LeuK1	111	194	222	435	564	649	762	875	960	1045	1173	675	562	477	392
LeuK2	111	194	222	435	564	649	762	875	960	1045	1173	487	374	289	204
LeuK3	111	194	222	435	564	649	762	875	960	1045	1173				

Supplementary Table 2. The pLDDT scores of six predicted active residues.

Residues	pLDDT score
D296	0.95
D321	0.95
D348	0.94
K363	0.95
D368	0.95
D395	0.92

Supplementary Table 3. RMSD values of LcsG and its homologies.

Name	PDB code	Pruned atom pairs		Overall	
		**pairs**	**RSMD (Å)**	**pairs**	**RSMD (Å)**
OxaC	5w7p	158	1.264	369	5.851
LepI	6ix7	124	0.946	372	6.219
ChOMT	1fp1	93	0.943	319	11.807
MmcR	3gwz	117	1.037	337	4.841
PhzM	2ip2	49	1.398	324	8.562
RedM	8tjj	84	1.424	324	5.692
RosA	4d7k	86	1.029	322	11.147

Supplementary Table 4. The system setup that includes number of simulations per system, simulation box dimensions, total number of atoms, total number of water molecules, salt concentration, liquid composition (number of molecules and type).

**Num of independent run**	**Box dimensions (Å)**	**Num of atoms**	**Num of water**	**Salt concentration**
3	88.124, 79.711, 74.777	52533	15062	0.15M

Supplementary Table 5. Known NMTs capable of furnishing at least two methyl transfers to at least one substrate and their similarity to LcsG.

Name	UniProt accessions numbers	Coverage /Identity
EgtD	A0R5M8	No significant similarity
PfPMT	Q8IDQ9	No significant similarity
BANMT	Q84N56	22/24.07
NRMT	Q9BV86	21/21.90
PsiM	P0DPA9	No significant similarity
RosA	K4RFM2	25/26.67
RedM	A0A0F7G196	27/29.13
OxyT	Q3S8P6	25/31.67
AMNMT	Q96565	31/24.07

Supplementary Table 6. Annotated enzymes clustered with LcsG in the sequence similarity network.

	**UniProt accessions numbers**	**Description**	**Involved in the biosynthesis of**	**Organism**	**Coverage /Identity**
OMT 1	Q0UI04	*O*-methyltransferase elcB	elsinochrome C	*Phaeosphaeria nodorum* strain SN15	88/37.27
OMT 2	P0CT89	4-*O*-methyltransferase 1	4-OH phenolic compounds	*Phanerochaete chrysosporium* strain RP-78	92/32.80
OMT 3	P0CT90	3-*O*-methyltransferase 2	3-OH phenolic compounds	*Phanerochaete chrysosporium* strain RP-78	88/33.04
OMT 4	A0A1U8QH20	*O*-methyltransferase cicE	cichorine	*Emericella nidulans* strain FGSC A4	90/33.88
OMT 5	Q0CCY5	*O*-methyltransferase gedA	geodin	*Aspergillus terreus* strain NIH 2624	73/29.41
OMT 6	Q4WQZ7	*O*-methyltransferase tpcA	trypacidin	*Aspergillus fumigatus* Af293	73/29.29
OMT 7	Q9UQY0	Demethylsterigmat-ocystin 6-*O*-methyltransferase	aflatoxins	*Aspergillus parasiticus* SU-1	85/34.23
OMT 8	I1RL18	*O*-methyltransferase dpfgI	pyrones	*Gibberella zeae*	81/22.43
OMT 9	A0A4P8WAD3	*O*-methyltransferase pyiA	pyrichalasin H	*Pyricularia grisea*	89/33.05
OMT 10	Q9P900	Demethylsterigmat-ocystin 6-*O*-methyltransferase	demethylsterigmatocystin, dihydrosterigmatocystin	*Aspergillus flavus* NRRL 3357	85/33.33
OMT 11	C9K2Q2	16-O-methyltransferase bsc6	brassicicene C	*Alternaria brassicicola*	82/33.33
OMT 12	S0DLP1	*O*-methyltransferase apf6	cyclic tetrapeptide apicidin F	*Gibberella fujikuroi* IMI 58289	81/24.3
OMT 13	B8NY85	*O*-methyltransferase agiB	aspergillicins	*Aspergillus flavus* NRRL 3357	71/35.11
OMT 14	B3FWS1	*O*-methyltransferase hmp5	hypothemycin	*Hypomyces subiculosus*	80/24.07
OMT 15	G3XSI5	*O*-methyltransferase aunD	aurasperone B	*Aspergillus niger*	98/34.62
OMT 16	I1RF60	*O*-methyltransferase aurJ	aurofusarin	*Gibberella zeae* PH-1	93/33.85
OMT 17	Q2I0M6	Dual *O*-methyltransferase/ FAD-dependent monooxygenase CTB3	cercosporin	*Cercospora nicotianae*	92/32.80
OMT 18	A2QBF0	*O*-methyltransferase aunD	aurasperone B	*Aspergillus niger* CBS 513.88	98/34.62
OMT 19	Q4WAW6	6-hydroxytryprostatin B *O*-methyltransferase	fumitremorgins	*Aspergillus fumigatus* Af293	91/37.07
OMT 20	S0DQQ0	*O*-methyltransferase fsr2	fusarubins	*Gibberella fujikuroi* IMI 58289	94/40.98
OMT 21	S0E608	*O*-methyltransferase bik3	bikaverin	*Gibberella fujikuroi* IMI 58289	80/47.57
OMT 22	Q0UI02	*O*-methyltransferase elcB	elsinochrome C	*Phaeosphaeria nodorum* SN15	82/37.27
OMT 23	D3H5H5	Chlorophenol *O*-methyltransferase	chlorophenol	Trichoderma longibrachiatum	89/42.98
OMT 24	A0A443HJY8	*O*-methyltransferase VdtC	viriditoxin	*Byssochlamys spectabilis*	93/46.77
OMT 25	L0MXX3	*O*-methyltransferase PaMT	fusicoccins	*Phomopsis amygdali*	94/41.18
OMT 26	D7PI16	*O*-methyltransferase gsfB	griseofulvin	*Penicillium aethiopicum*	80/47.57

OMT 27	A0A1U9YI02	*O*-methyltransferase verK	11'-deoxyverticillin A	*Clonostachys rogersoniana*	99/26.87
OMT 28	Q2UPA6	*O*-methyltransferase aclU	aspirochlorine	*Aspergillus oryzae* RIB 40	92/22.48
OMT 29	Q2UPB3	*O*-methyltransferase aclM	aspirochlorine	*Aspergillus oryzae* RIB 40	86/30.71
OMT 30	Q4WMJ5	*O*-methyltransferase gliM	gliotoxin	*Aspergillus fumigatus* Af293	86/28.81
OMT 31	P55790	Sterigmatocystin 8-*O*-methyltransferase	sterigmatocystin	*Aspergillus flavus* NRRL 3357	84/33.33
OMT 32	Q12120	Sterigmatocystin 8-*O*-methyltransferase	sterigmatocystin	*Aspergillus parasiticus* SU-1	84/34.26
OMT 33	A0A142C7A1	*O*-methyltransferase phnC	atrovenetin	*Penicillium herquei*	79/22.94
OMT 34	D7PI17	*O*-methyltransferase gsfC	griseophenone D	*Penicillium aethiopicum*	79/27.45
OMT 35	D7PI18	*O*-methyltransferase gsfD	desmethyl-dehydrogriseofulvin	*Penicillium aethiopicum*	85/31.25
OMT 36	A0A067Z9B6	*O*-methyltransferase af390-400	fumagillin	*Aspergillus fumigatus* Af293	25/32.81
OMT 37	A0A0C1E5J2	*O*-methyltransferase opaF	oxepinamides	*Aspergillus ustus*	40/31.48
OMT 38	A0A0B5L781	*O*-methyltransferase mpaG	mycophenolic acid	*Penicillium brevicompactum*	94/33.87
OMT 39	B8N8R1	*O*-methyltransferase afvC	aflavarin	*Aspergillus flavus* NRRL 3357	81/26.79
EqxD	S4W780	Methyltransferase eqxD	equisetin	*Fusarium heterosporum*	78/26.92
Fsa4	A0A0E3VJW8	Methyltransferase fsa4	fusarisetin A	*Fusarium* sp. strain FN080326	81/26.17
PynC	A5ABG3	Methyltransferase pynC	pyranonigrins	*Aspergillus niger* CBS 513.88	90/28.24
Phm5	A0A2Z5XAK6	Methyltransferase phm5	phomasetin	*Pyrenochaetopsis* sp.	83/29.46
NanE	A0A6G9KJC3	*N*-methyltransferase nanE	nanangelenin	*Aspergillus nanangensis*	82/31.13

Supplementary Table 7. Non-ribosomal peptide synthetases (NRPSs) contained terminal modules similar to that of LcsG.

**Name**	**UniProt Accession No.**	**Gene cluster**	**Organism**	**Coverage /Identity**
InpA	Q5B7I5	Fellutamide B	*Aspergillus nidulans* FGSC A4	100/41.57
ApmA	A0A1W6BT53	Asperphenamate	*Penicillium brevicompactum*	100/42.35
AtnA	Q5AUZ6	Aspercryptin	*Aspergillus nidulans* FGSC A4	100/42.16
NRPS 5	I1SAJ7	Fusaoctaxin A	*Fusarium graminearum* PH-1	92/40.69
PpzA	A0A166YZW0	Pyrrolopyrazine	*Metarhizium rileyi* RCEF 4871	100/40.24
PerA	Q4H424	Peramine	*Epichloe festucae* Fl1	96/41.49
LgrD	Q70LM4	Linear gramicidin	*Brevibacillus parabrevis*	89/33.62

Supplementary Table 8. Plasmids and strains used in this study.

**Plasmids/Strains**	**Description**	**Reference**
pKOV21	geneticin (*neo*, G418) resistance vector, Amp	Shen, B. et al. Development of a high-efficiency gene knockout system for *Pochonia chlamydosporia*
pEASY	*LacZα*, T7 promoter, Kan^r, Amp	TransGen Biotech
pACYCDuet-1	*lacI*, T7 promoter-1, T7 promoter-2, Cm^r	Novagen
KSTNP	*TrpC* terminator and *neo* contained vector	Wang, G. et al. Biosynthesis of Antibiotic Leucinostatins in Bio-control Fungus *Purpureocillium lilacinum* and Their Inhibition on *Phytophthora* Revealed by Genome Mining
PCH-sGFP	*gpdA(p)* contained vector	Liang, L. et al. A high efficiency gene disruption strategy using a positive– negative split selection marker and electroporation for *Fusarium oxysporum*
pGNT	*neo*, *TrpC*, *gpdA(p)* in pEASY	This study
pKOV21-ko*lcsG*	*lcsG* deletion cassette in pKOV21	This study
pGNT-*lcsG*	*lcsG* overexpression cassette in pGNT	This study
pACYC-*lcsG*	*lcsG* expression cassette in pACYCDuet-1	This study
Purpureocillium lilacinum strain PLBJ-1 (CGMCC3.17492)	Wild type	This study
Escherichia coli DH5α		Tsingke
Escherichia coli ArcticExpress (DE3)	*E. coli* B F$^-$ *ompT hsdS*(r_B^- m_B^-) *dcm*$^+$ Tet^r *gal* λ(DE3) *endA* Hte [*cpn10 cpn60* Gent^r]	Agilent Technologies
PLBJ-Δ*lcsG*	Δ*lcsG*::*neo* in *P. lilacinum* PLBJ-1	This study
PLBJ-OE*lcsG*	*neo, gpdA*::*lcsG*::*TrpC* in *P. lilacinum* PLBJ-1	This study
E. coli ArcticExpress (DE3)-*lcsG*	*E. coli* ArcticExpress (DE3) pACYC-*lcsG*	This study

Supplementary Table 9. Primers used in this study.

Primers name	Sequence (5′ to 3′)
*lcsG*up-f	TCCCCGCGGAAGTCGGGTAGACAGGGTGAAAT
*lcsG*up-r	AAGGAAAAAAGCGGCCGCACAGATGAAGCTGCGTTTACGAC
*lcsG*down-f	GACTAGTGCTGCGATAGTCGCTACTAAAGC
*lcsG*down-r	CGCGGATCCGCCTTGAGCAACATTCTGGC
lcsGup-ne-r	CGTAAAGCACGAGGAAGCG
eo-lcsGdown-f	GACTGGGCACAACAGACAATCG
p*lcsG*KO SCR-f	AAGCTCATCAAACGGTCAAACAT
p*lcsG*KO SCR-r	CACTCACCAAGTCAGTGCTCCTC
*lcsG*check-f	CCCTGAAAGAGCGACTGGAG
*lcsG*check-r	GGCTCAAAGATGCGGTTGCTA
QC-*lcsG*-f	gatataccatgggcagcagccatcaccatcatcaccacATGGGAGACAACGTTCAGTCC
QC-*lcsG*-r	gcgccgagctcgaattcggatcctggctTCAGTGGTGGTGGTGGTGGTGGCCACGCCAAAC<br>AGCCTC<br>GA
pEASY-*neoTtrpC*-f	ttaattaagtttaaacACTTAACGTTACTGAAATCATCAAA
pEASY-*neoTtrpC*-r	TCTAGATTAACGCTTACAATTTCCA
pGNT-*gpdA*-f	CATGgggcccGCCATTCAGGCTGCGCAACTGTTGG
Pgnt-*gpdA*-r	AAGGAAAAAAgcggccgcGGTGATGTCTGCTCAAGCGGGGTAG
OE-*lcsG*-f	AGCAGACATCACCGCATGGGAGACAACGTTCAGTC
OE-*lcsG*-r	CAGTAACGTTAAGTgCTAGCCACGCCAAACAGCCTC
OE-*lcsG*-SCR-f	GTTGACAAGGTCGTTGCGTC
OE-*lcsG*-SCR-r	GATAGCCTCAACCGCCTCCTG
Actin-f	GCCCTCTGTCCTGGGTCTT
Actin-r	ACAGGGAGGCGAGAATGGA
qp-*lcsG*-f	CTCGTGTCCTCGTCCTGGATCTG
qp-*lcsG*-r	GATCATCCGCTGCCTTGAAGAGATC
mutant-D296-F	CAAAGCCACCGTCGTTGcTCTTGGTGGCTCTGGC
mutant-D296-R	GCCAGAGCCACCAAGAgCAACGACGGTGGCTTTG
mutant-D321-F	CAAGATCATCGTCCAGGcTCTGCCTAGTTGCCAA
mutant-D321-R	TTGGCAACTAGGCAGAgCCTGGACGATGATCTTG
mutant-D348-F	TCCTTCCTCGCTCATGcCTTCTTCACCCCTCAG
mutant-D348-R	CTGAGGGGTGAAGAAGgCATGAGCGAGGAAGGA
mutant-K363-F	CGACATCTACCTCTTCgcGTGGGTCTTTTACGACT
mutant-K363-R	AGTCGTAAAAGACCCACgcGAAGAGGTAGATGTCG
mutant-Y367-F	CTTCAAGTGGGTCTTTgcCGACTGGTCCAACAAG
mutant-Y367-R	CTTGTTGGACCAGTCGgcAAAGACCCACTTGAAG
mutant-D368-F	CAAGTGGGTCTTTTACGcCTGGTCCAACAAGGAC
mutant-D368-R	GTCCTTGTTGGACCAGgCGTAAAAGACCCACTTG
mutant-D395-F	CGTGTCCTCGTCCTGGcTCTGATGGTGGACGTC
mutant-D395-R	GACGTCCACCATCAGAgCCAGGACGAGGACACG
mutant-K431-F	GCTGTTTGGACATACCgcGCAGGCGACGAAGAAG
mutant-K431-R	CTTCTTCGTCGCCTGCgcGGTATGTCCAAACAGC

Supplementary Figures

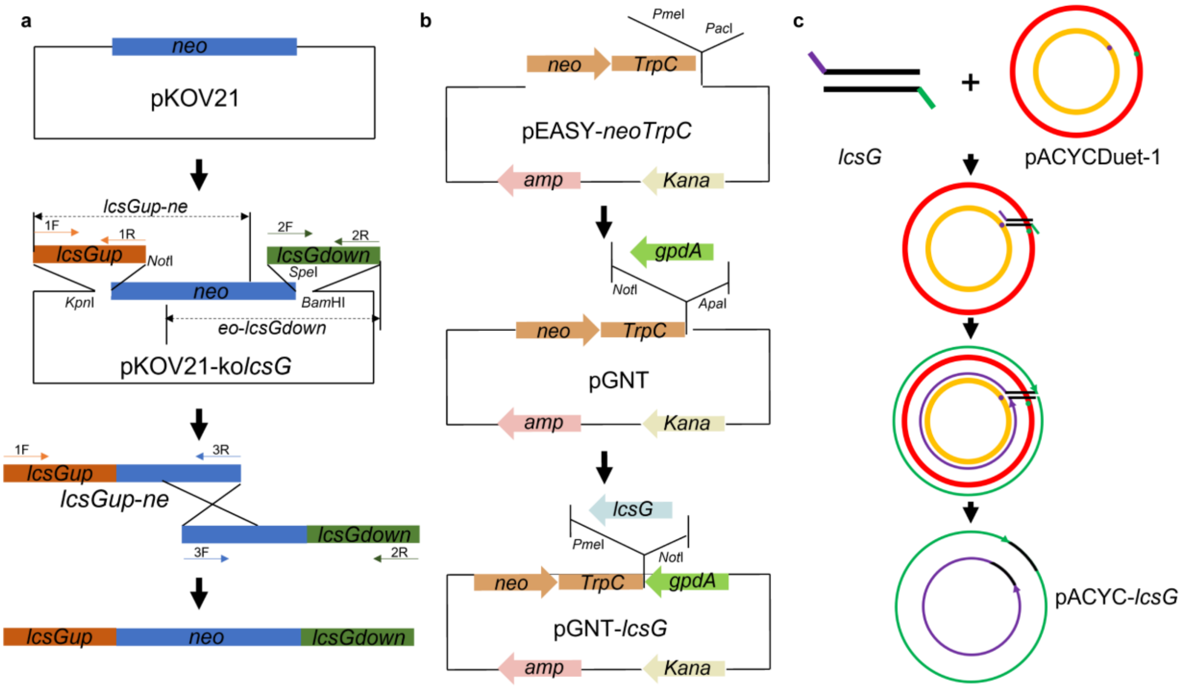

Supplementary Figure 1. The scheme of plasmids construction. **a)** Construction of the *lcsG* deletion vector pKOV21-ko*lcsG,* and schematic diagram of split-mark to disrupt the gene. 1F: *lcsG*up-f, 1R: *lcsG*up-r; 2F: *lcsG*down-f, 2R: *lcsG*down-r; 3R: *lcsGup-ne*-r 3F: *eo-lcsGdown*-f. **b)** Construction of the *lcsG* overexpression vector pGNT-*lcsG*. **c)** Construction of the recombinant protein LcsG expression vector pACYC-*lcsG*. The recombinant plasmid was constructed by using the quick-change method.

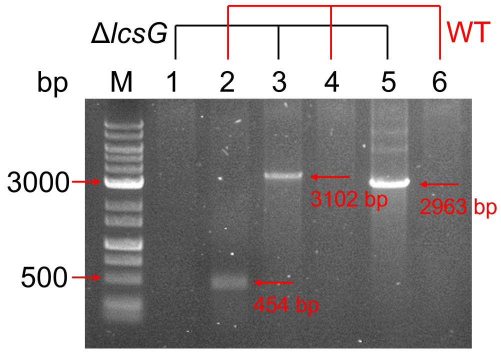

Supplementary Figure 2. Confirmation of the *lcsG* deletions in *P. lilacinum* strain PLBJ-1. Marker was FastGene 1 kb DNA Marker Plus (NIPPON Genetics). Lane 1, 3, 5 showed PCR products from the *lcsG* knock-out strain. Lane 2, 4, 6 showed PCR products from PLBJ-1 (WT). Lane 1, 2 were amplified with primer pair *lcsG* check-f and *lcsG* check-r. Lane 3, 4 were amplified with primer pair *lcsG* up-f and *lcsGup-ne*-r. Lane 5, 6 were amplified with primer pair *eo-lcsGdown*-f and *lcsG*down-r.

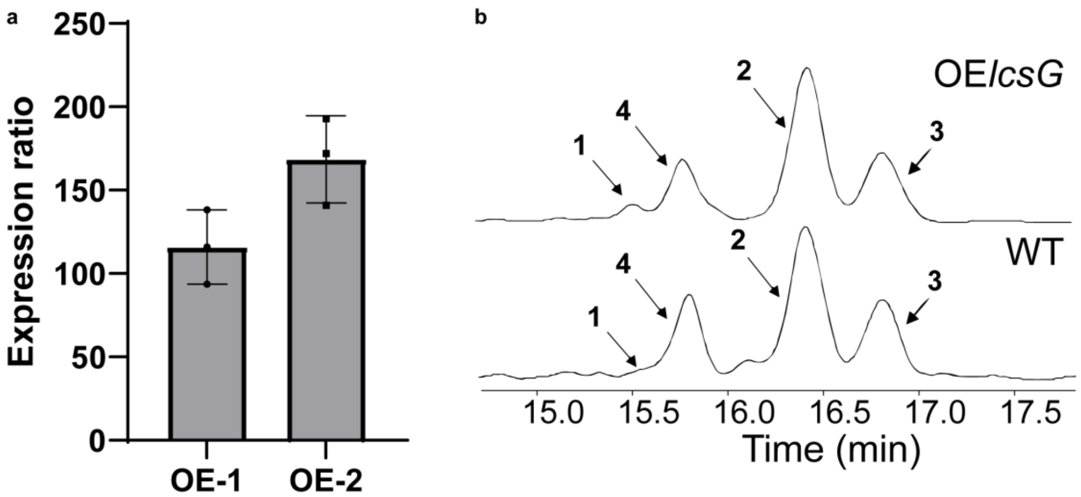

Supplementary Figure 3. a) Quantitative analysis of *lcsG* gene expression in overexpression (OE) mutants by quantitative real- time PCR. The gene expression level of *lcsG* in OE-1 and OE-2 demonstrate 115.69-fold and 168.56-fold upregulation, respectively. All data represent the mean of n = 3 biologically independent samples and error bars show standard deviation. **b)** LC-MS analysis of leucinostatin C (LeuC, **1**), LeuB (**2**), LeuA (**3**), and LeuK0 (**4**) of the *lcsG* overexpression (OE*lcsG*) mutant and WT.

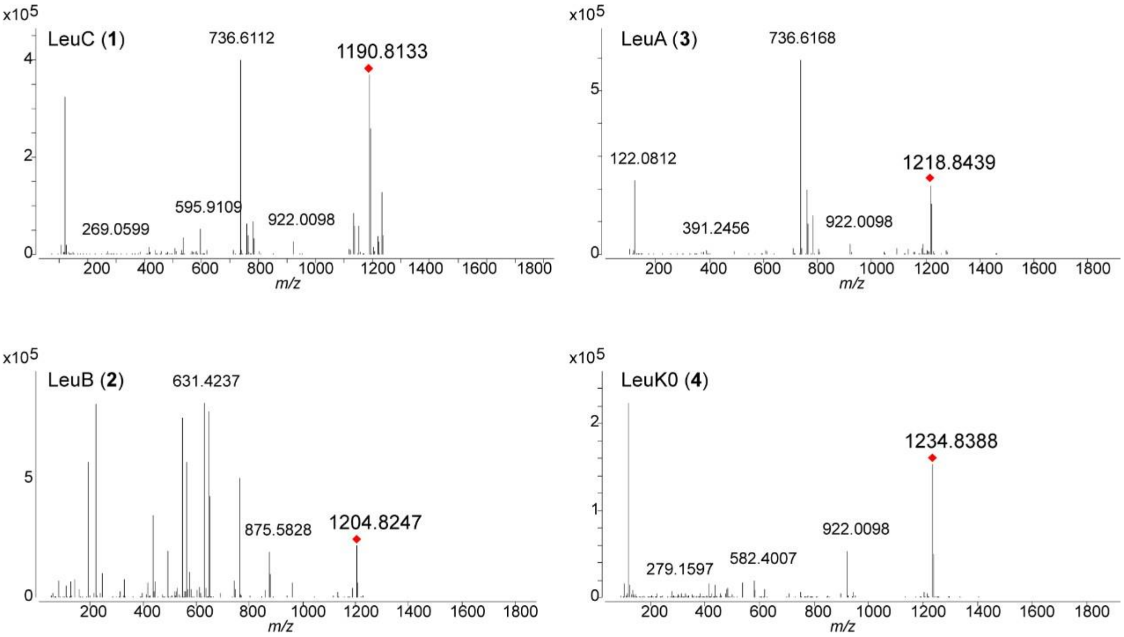

Supplementary Figure 4. HRESI-MS spectrum of LeuC (**1**), LeuB (**2**), LeuA (**3**), and LeuK0 (**4**).

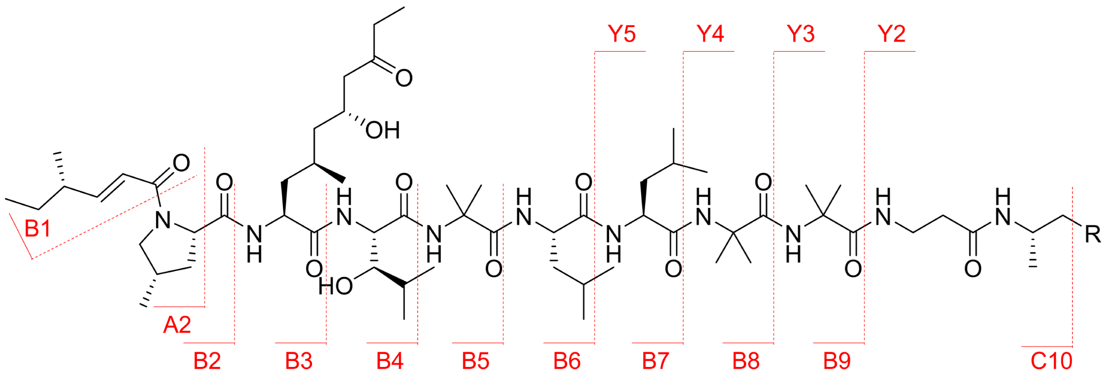

Supplementary Figure 5. Fragmentation types observed in MS-MS spectra of leucinostatins.

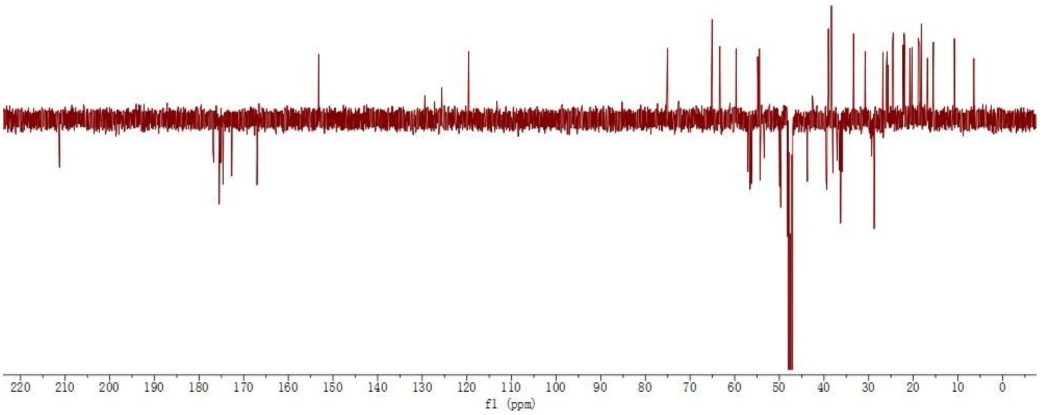

Supplementary Figure 6. ^{13}C-NMR (APT) of LeuK0 in CD_3OD. It revealed that LeuK0 possessed total of 62 carbons, including 18 methyl groups (-CH3), 14 methylenes (-CH2), 16 methines (-CH), and 14 sp3 quaternary carbons.

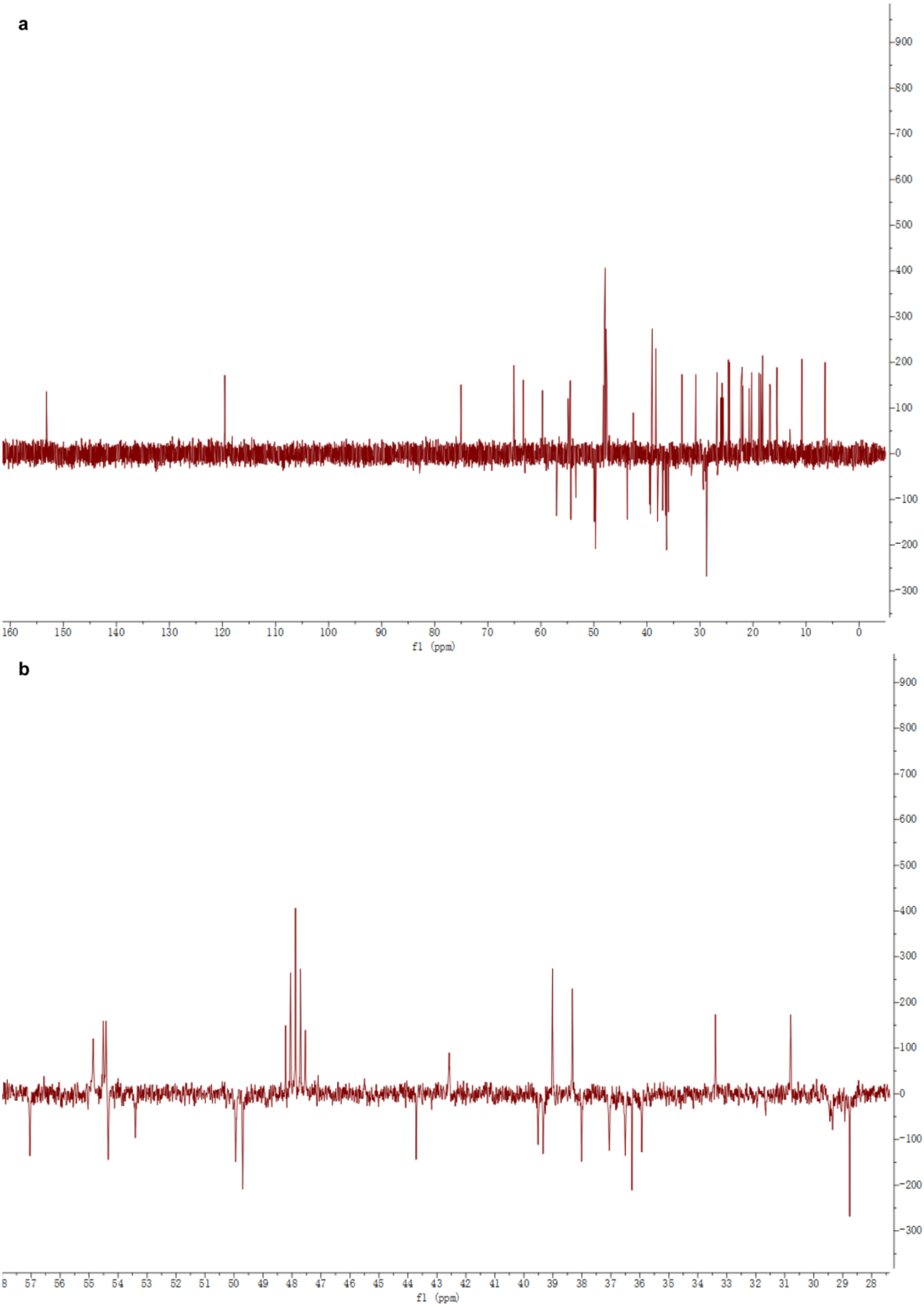

Supplementary Figure 7. ^{13}C-NMR (DEPT-135) of LeuK0 in CD_3OD. **a)**The global view of this spectrum. **b)** The local view of this spectrum. The 13C-NMR DEPT-135 data of LeuK0 indicated it contains 14 -CH2 units and 34 -CH/CH3 units.

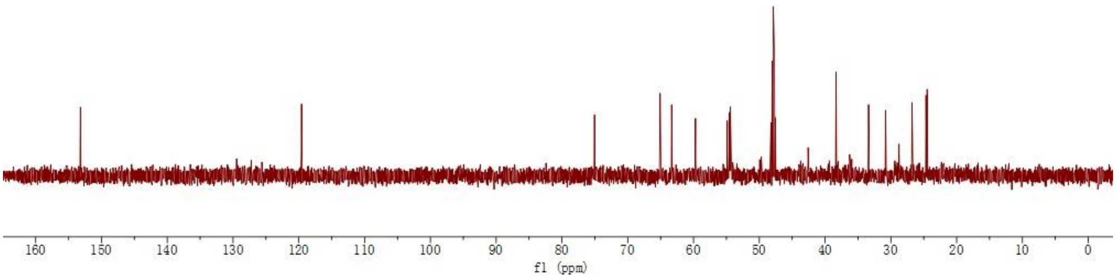

Supplementary Figure 8. ^{13}C-NMR (DEPT-90) of LeuK0 in CD_3OD. The 13C-NMR DEPT-90 spectrum of LeuK0 indicated it contains 16 -CH units.

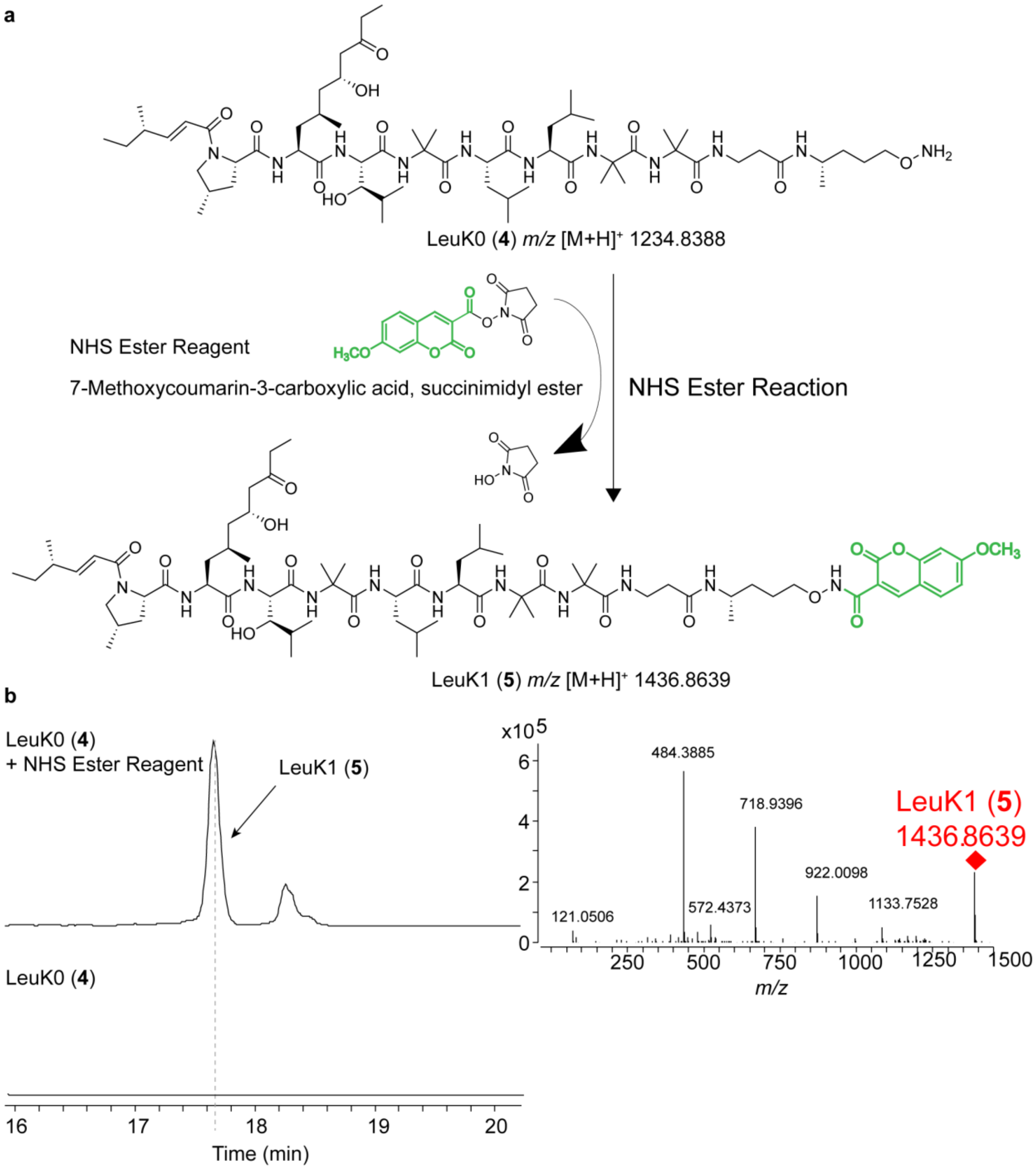

Supplementary Figure 9. NHS ester reaction scheme. **a)** NHS ester reaction scheme for chemical conjugation to LeuK0 (**4**). 7-Methoxycoumarin-3-carboxylic acid succinimidyl ester was employed as the NHS ester reagent. **b)** LC- MS analysis of LeuK0 (**4**) and NHS ester reaction with LeuK0. EIS at *m/z* 1236.8469 refers to $[M+H]^+$ ion of product LeuK1 (**5**).

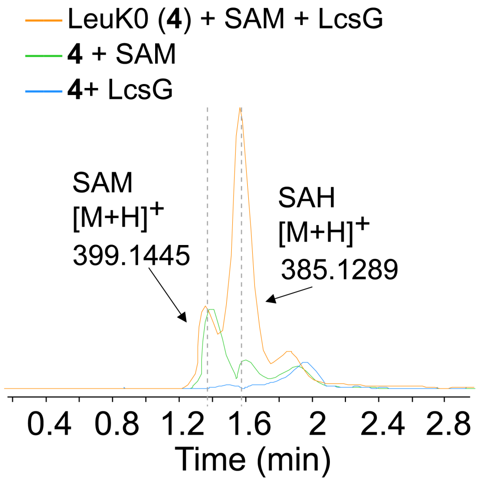

Supplementary Figure 10. LC-MS detection of SAM and SAH of LcsG-catalyzed reactions.

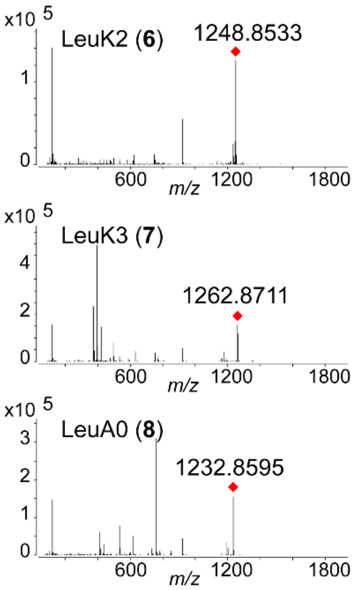

Supplementary Figure 11. $[M+H]^+$ ions of LeuK2 (**6**), LeuK3 (**7**) and LeuA0 (**8**).

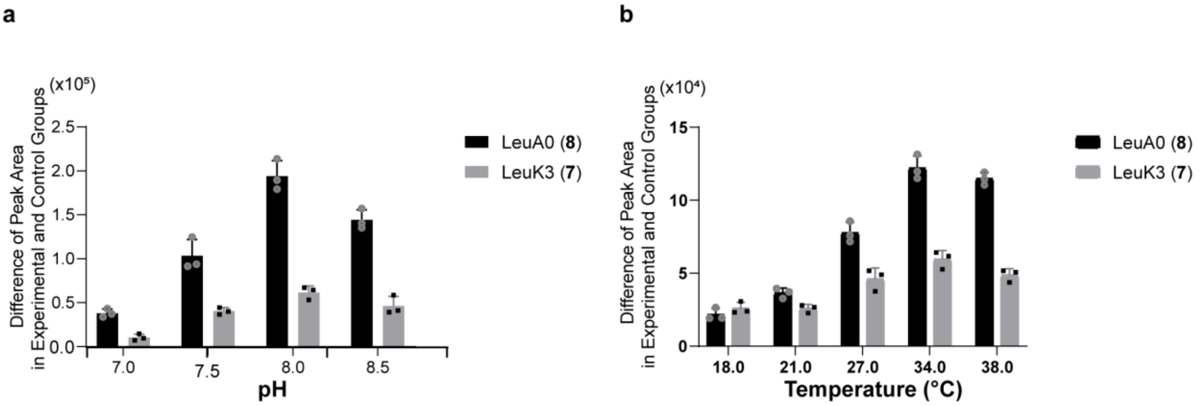

Supplementary Figure 12. a) Effect of pH (pH 7.0, pH 7.5. pH 8.0, pH 8.5) on the production of LeuA0 (**8**) and LeuK3 (**7**) by LcsG- catalyzed. LcsG showed a pH optimum of 8.0 (labeled in red). **b)** Effect of temperature (18°C, 21°C, 27°C, 34°C, 38°C) on the production of LeuA0 (**8**) and LeuK3 (**7**) by LcsG-catalyzed. LcsG showed a temperature optimum of 34 °C (labeled in blue). All data represent the mean of n = 3 biologically independent samples and error bars show standard deviation (a, b).

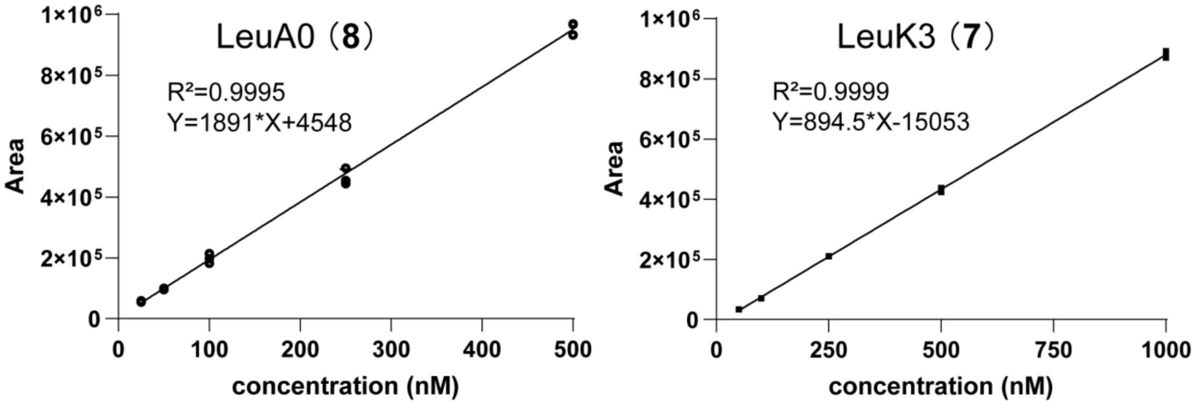

Supplementary Figure 13. Standard curve of concentration and peak area of LeuA0 (**8**) and LeuK3 (**7**) determined by LC-MS. All data represent the mean of n = 3 biologically independent samples and error bars show standard deviation.

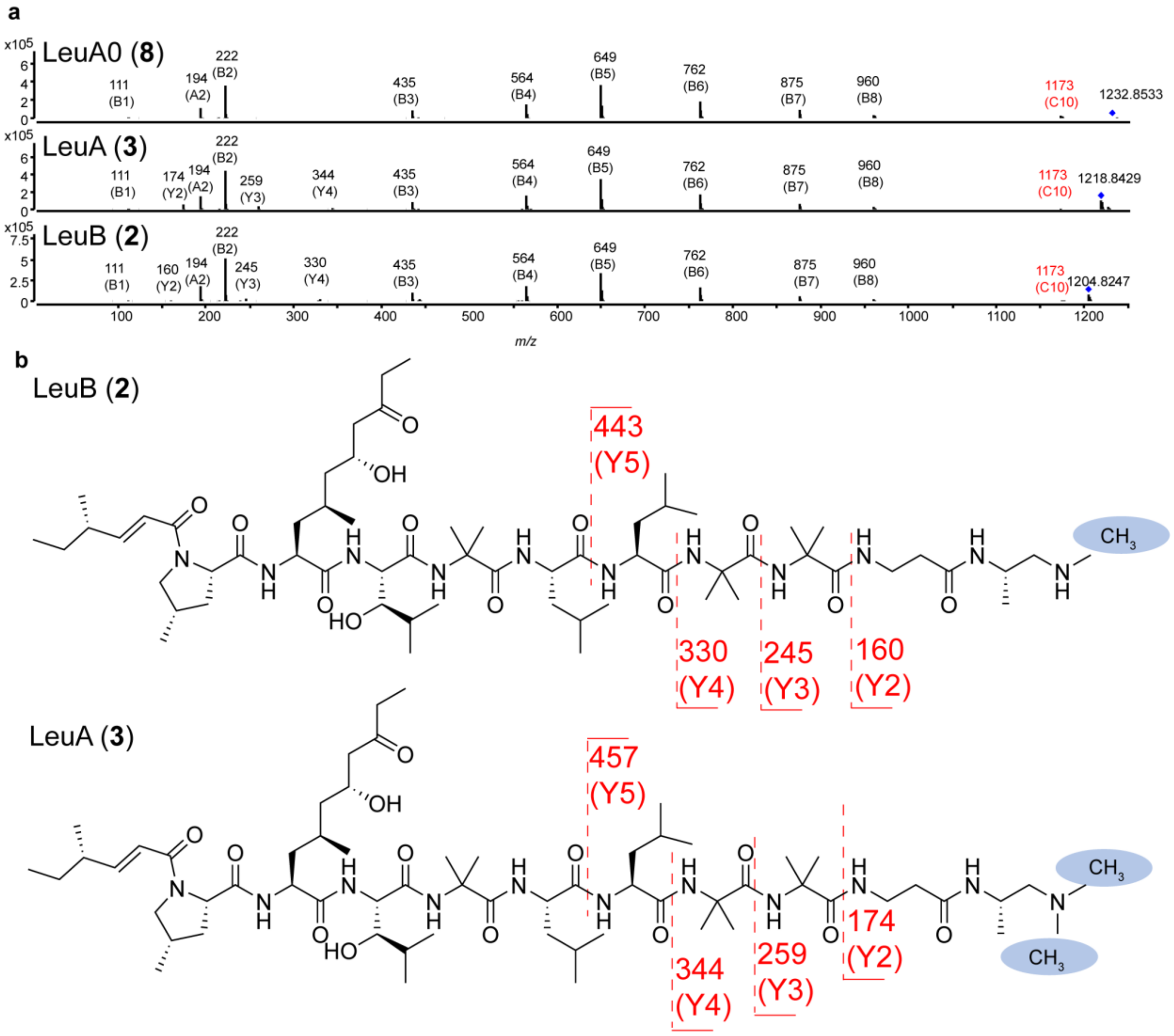

Supplementary Figure 14. a) Ions from the HRESI-MS-MS data of LeuB (**2**), LeuA (**3**), and LeuA0 (**8**). **b)** Predicted fragments and their calculated *m/z* for observed ions got from HRESI- MS-MS of LeuB (**2**), LeuA (**3**).

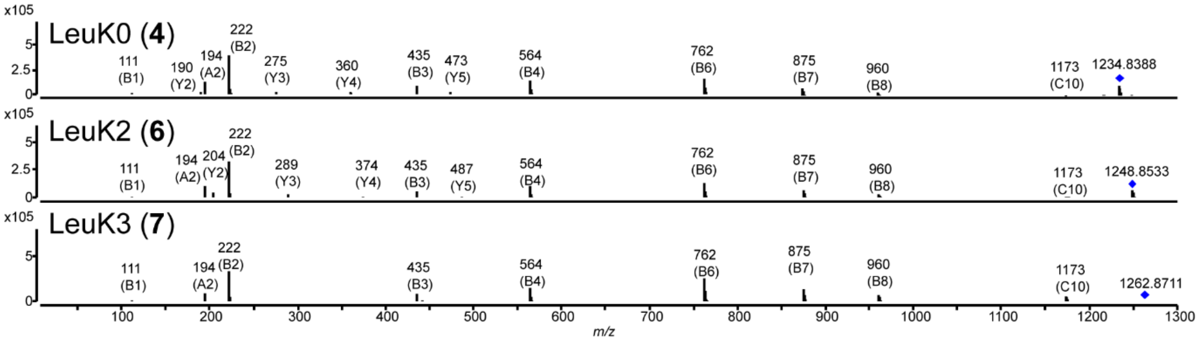

Supplementary Figure 15. Ions from the HRESI-MS-MS data of LeuK0 (**4**), LeuK2 (**6**), and LeuK3 (**7**).

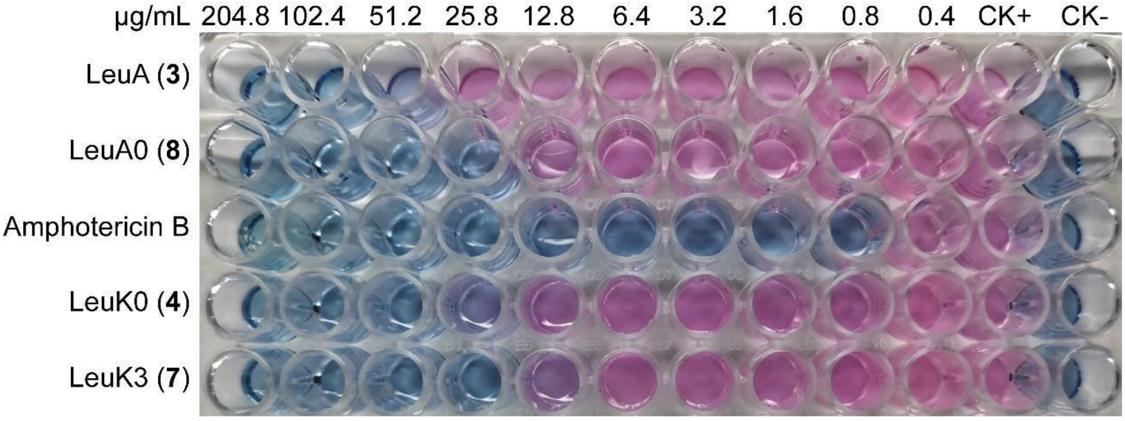

Supplementary Figure 16. MIC (μg/mL) of leucinostatins (LeuA (**3**), LeuA0 (**8**), LeuK0 (**4**), LeuK3 (**7**)) against *Cryptococcus neoformans* strain H99 in a 2-fold liquid dilution assay. The 2-fold serial dilution of leucinostatins from 204.8 to 0.4 μg/mL was tested in 96-well plates. The positive control was amphotericin B at a 2-fold serial dilution from 204.8 to 0.4 μg/mL. The negative controls were medium (CK-) and inoculum (CK+).

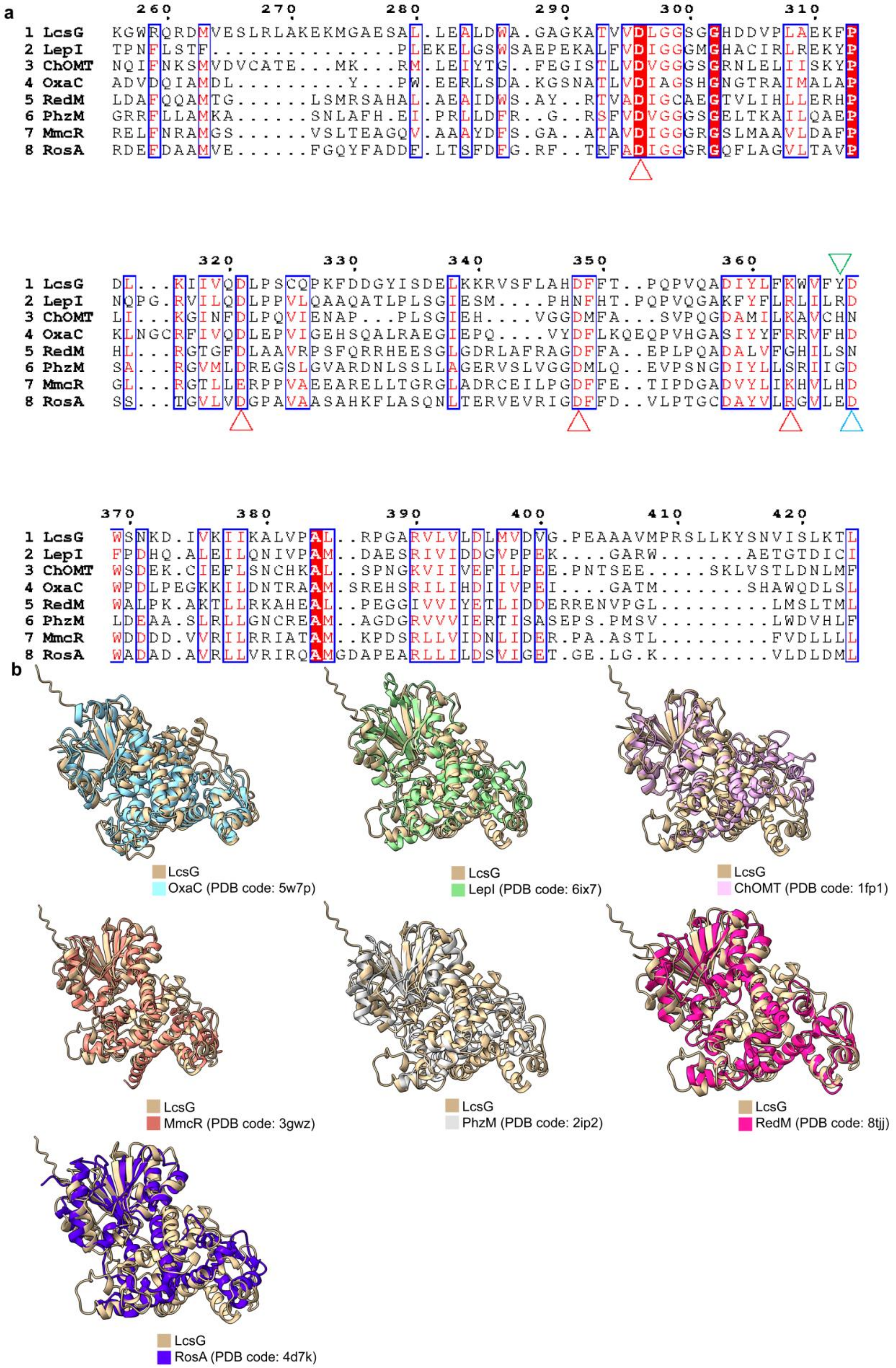
a
260 270 280 290 300 310
1 LcsG KGWRQRDMVESLRLAKEKMGAESAL.LEALDWA.GAGKATVVDLGGSGGHDDVPLAEKFP
2 LepI TPNFLSTF..............PLEKELGSWSAEPEKALFVDIGGGMGHACIRLREKYP
3 ChOMT NQIFNKSMVDVCATE...MK...RM.LEIYTG..FEGISTLVDVGGGSGRNLELIISKYP
4 OxaC ADVDQIAMDL.........Y...PW.EERLSDA.KGSNATLVDIAGSHGNGTRAIMALAP
5 RedM LDAFQQAMTG......LSMRSAHAL.AEAIDWS.AY..RTVADIGCAEGTVLIHLLERHP
6 PhzM GRRFLLAMKA......SNLAFH.EI.PRLLDFR..G..RSFVDVGGGSGELTKAILQAEP
7 MmcR RELFNRAMGS......VSLTEAGQV.AAAYDFS.GA..ATAVDIGGGRGSLMAAVLDAFP
8 RosA RDEFDAAMVE......FGQYFADDF.LTSFDFG.RF..TRFADIGGGRGQFLAGVLTAVP
320 330 340 350 360
1 LcsG DL...KIIVQDLPSCQPKFDDGYISDELKKRVSFLAHDFFT..PQPVQADIYLFKWVFYD
2 LepI NQPG.RVILQDLPPVLQAAQATLPLSGIESM....PHNFHT.PQPVQGAKFYFLRLILRD
3 ChOMT LI...KGINFDLPQVIENAP...PLSGIEH....VGGDMFA...SVPQGDAMILKAVCHN
4 OxaC KLNGCRFIVQDLEPVIGEHSQALRAEGIEPQ....VYDFLKQEQPVHGASIYYFRRVFHD
5 RedM HL...RGTGFDLAAVRPSFQRRHEESGLGDRLAFRAGDFFA..EPLPQADALVFGHILSN
6 PhzM SA...RGVMLDREGSLGVARDNLSSLLAGERVSLVGGDMLQ..EVPSNGDIYLLSRIIGD
7 MmcR GL...RGTLLERPPVAEEARELLTGRGLADRCEILPGDFFE..TIPDGADVYLIKHVLHD
8 RosA SS...TGVLVDGPAVAASAHKFLASQNLTERVEVRIGDFFD..VLPTGCDAYVLRGVLED
370 380 390 400 410 420
1 LcsG WSNKD.IVKIIKALVPAL..RPGARVLVLDLMVDVG.PEAAAVMPRSLLKYSNVISLKTL
2 LepI FPDHQ.ALEILQNIVPAM..DAESRIVIDDGVPPEK....GARW.......AETGTDICI
3 ChOMT WSDEK.CIEFLSNCHKAL..SPNGKVIIVEFILPEE.PNTSEE.....SKLVSTLDNLMF
4 OxaC WPDLPEGKKILDNTRAAM.SREHSRILIHDIIVPEI....GATM.......SHAWQDLSL
5 RedM WALPK.AKTLLRKAHEAL..PEGGIVVIYETLIDDERRENVPGL.........LMSLTML
6 PhzM LDEAA.SLRLLGNCREAM..AGDGRVVVIERTISASEPS.PMSV.........LWDVHLF
7 MmcR WDDDD.VVRILRRIATAM..KPDSRLLVIDNLIDER.PA.ASTL.........FVDLLLL
8 RosA WADAD.AVRLLVRIRQAMGDAPEARLLILDSVIGET.GE.LG.K.........VLDLDML
b
LcsG
OxaC (PDB code: 5w7p)
LcsG
LepI (PDB code: 6ix7)
LcsG
ChOMT (PDB code: 1fp1)
LcsG
MmcR (PDB code: 3gwz)
LcsG
PhzM (PDB code: 2ip2)
LcsG
RedM (PDB code: 8tjj)
LcsG
RosA (PDB code: 4d7k)

Supplementary Figure 17. Comparison between the LcsG and its structurally characterized homologues. ChOMT (UniProt accessions number: P93324), OxaC (UniProt accessions number: A0A1B2TT09), MmcR (UniProt accessions number: Q9X5T6) were characterized as OMTs; RedM (UniProt accessions number: O54154), PhzM (UniProt accessions number: Q9HWH2), and RosA (UniProt accessions number: K4RFM2) were characterized as NMTs; LepI (UniProt accessions number: B8NJH3) was characterized as a SAM-dependent OMT-like pericyclase-dehydratase. **a)** Sequence comparison between the LcsG and these homologues. White letters on a red background indicated strictly conserved amino acid residues. Red letters in blue boxes indicated well-conserved amino acids or similar amino acids. The positions marked with red triangles are predicted to be part of the SAH binding motif; those labeled with blue triangles are predicted catalytic residues; the green triangle indicated LcsG's Y367 and its corresponding residues. **b)** Structural comparison between LcsG and these homologies.

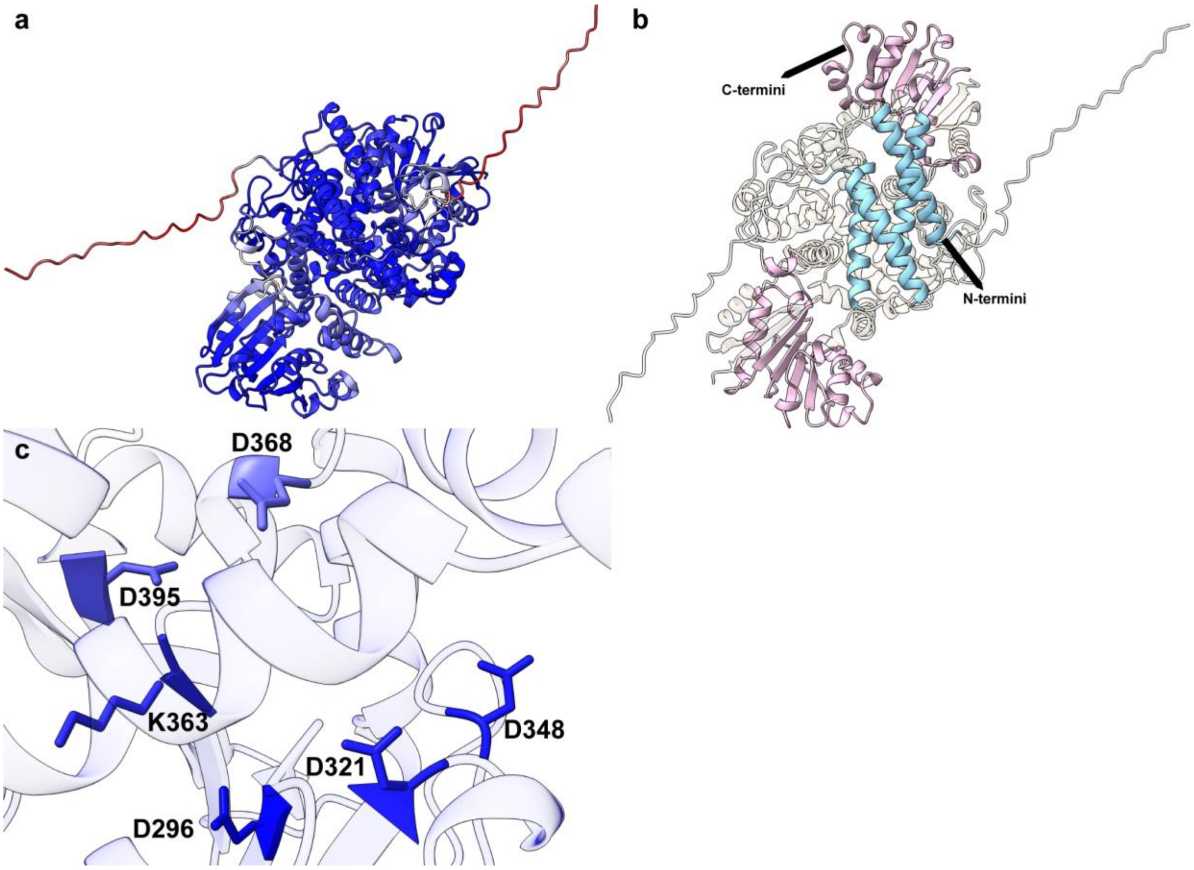

Supplementary Figure 18. a) The overall structure of LcsG with color-coded pLDDT scores, where a blue hue corresponds to a pLDDT score of 1.0, indicating high confidence, while a red hue signifies a pLDDT score of 0, representing lower confidence. **b)** Cartoon representation of LcsG dimer. The C-terminal acceptor binding site and the N-terminal helices were depicted in pink and blue, respectively. **c)** The pLDDT scores of the six predicted active site residues.

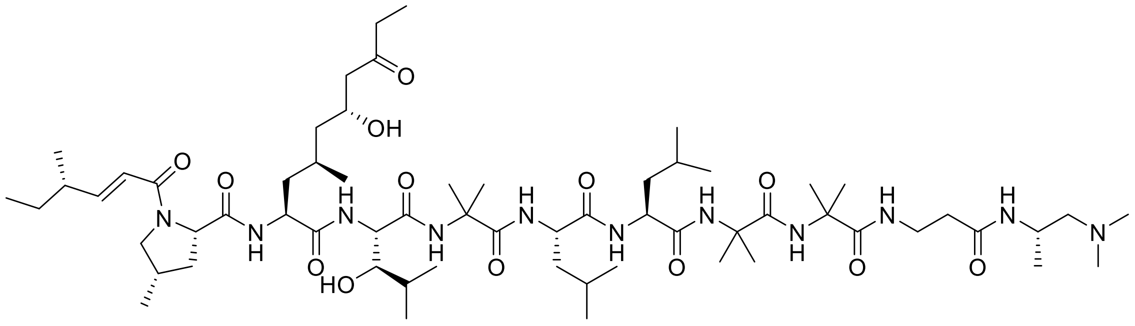

LeuA

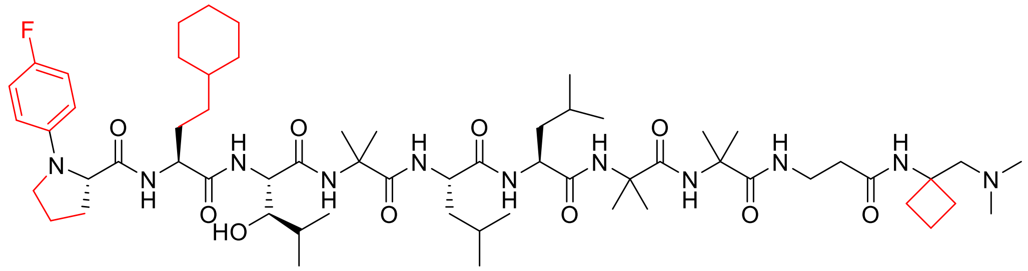

ZHAWOC6027

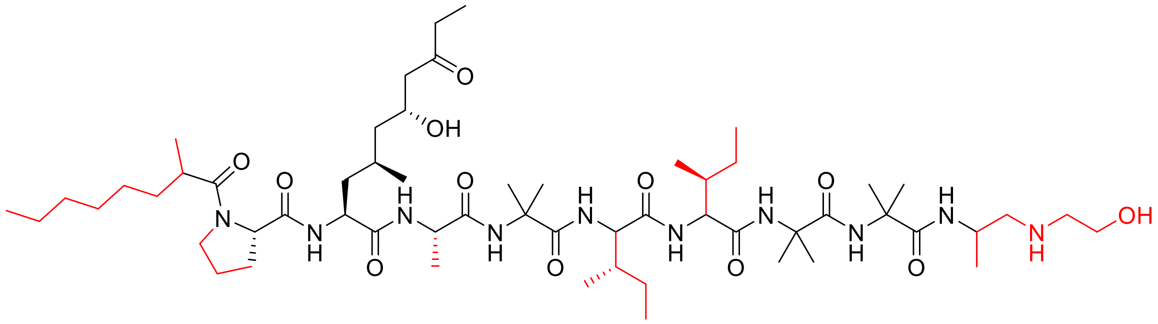

helioferin A

Supplementary Figure 19. Formulas and sequences of LeuA, ZHAWOC6027, and helioferin A. Structural motifs that are different from LeuA are shown in red.

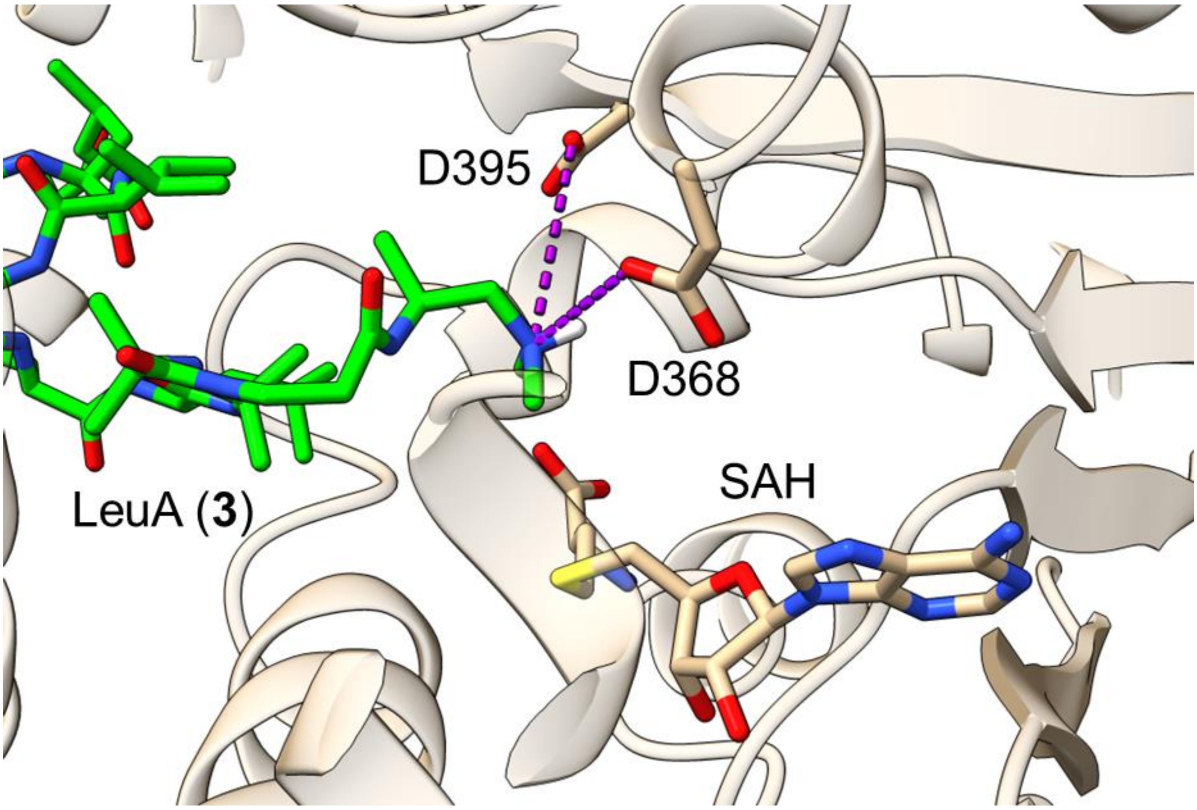

Supplementary Figure 20. The results of Diffdock analysis utilizing the modified LeuA structure and SAH.

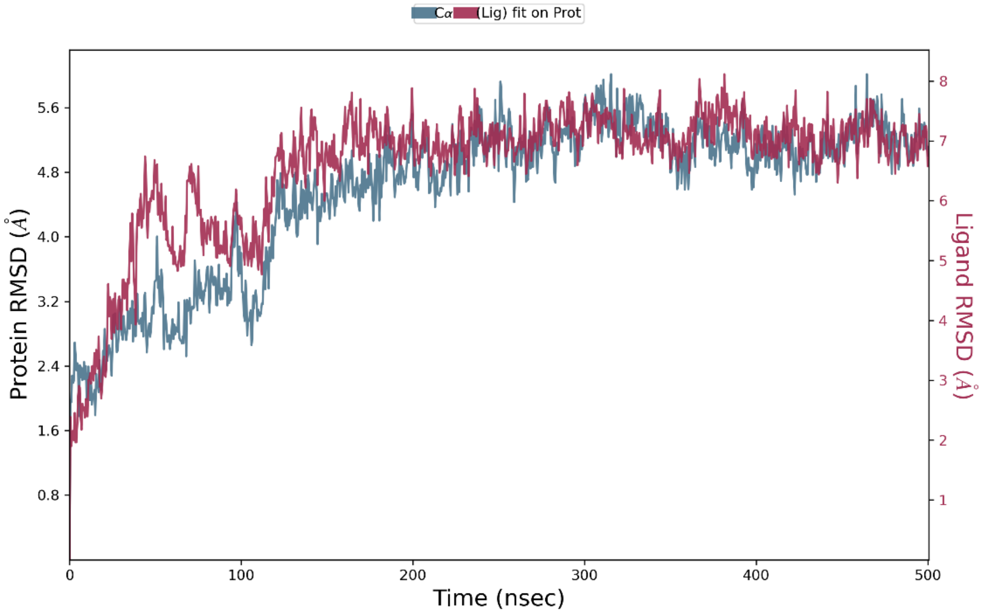

Supplementary Figure 21. 500 ns trajectories protein & ligand RMSD. Protein and ligand were stable after 500 ns MD simulation.

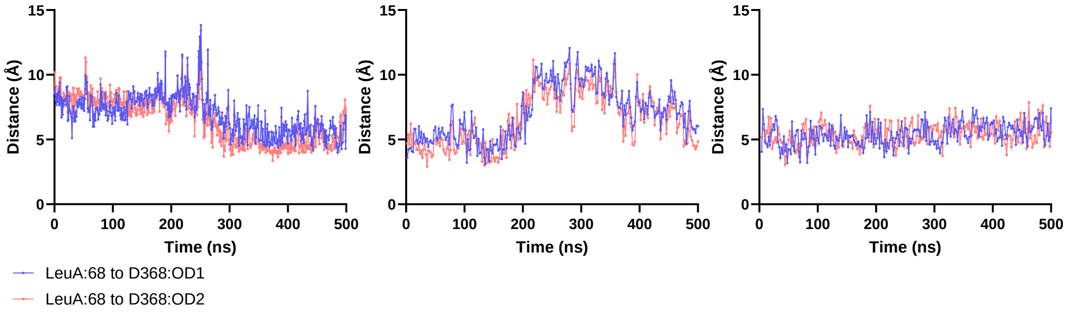

Supplementary Figure 22. Interatomic distance between targeted N of LeuA (LeuA:68) and two O of D368 (OD1 and OD2) in 3 independent MD simulations. Following a 500 ns equilibration period, three additional independent simulations, each lasting 500 ns, were conducted.

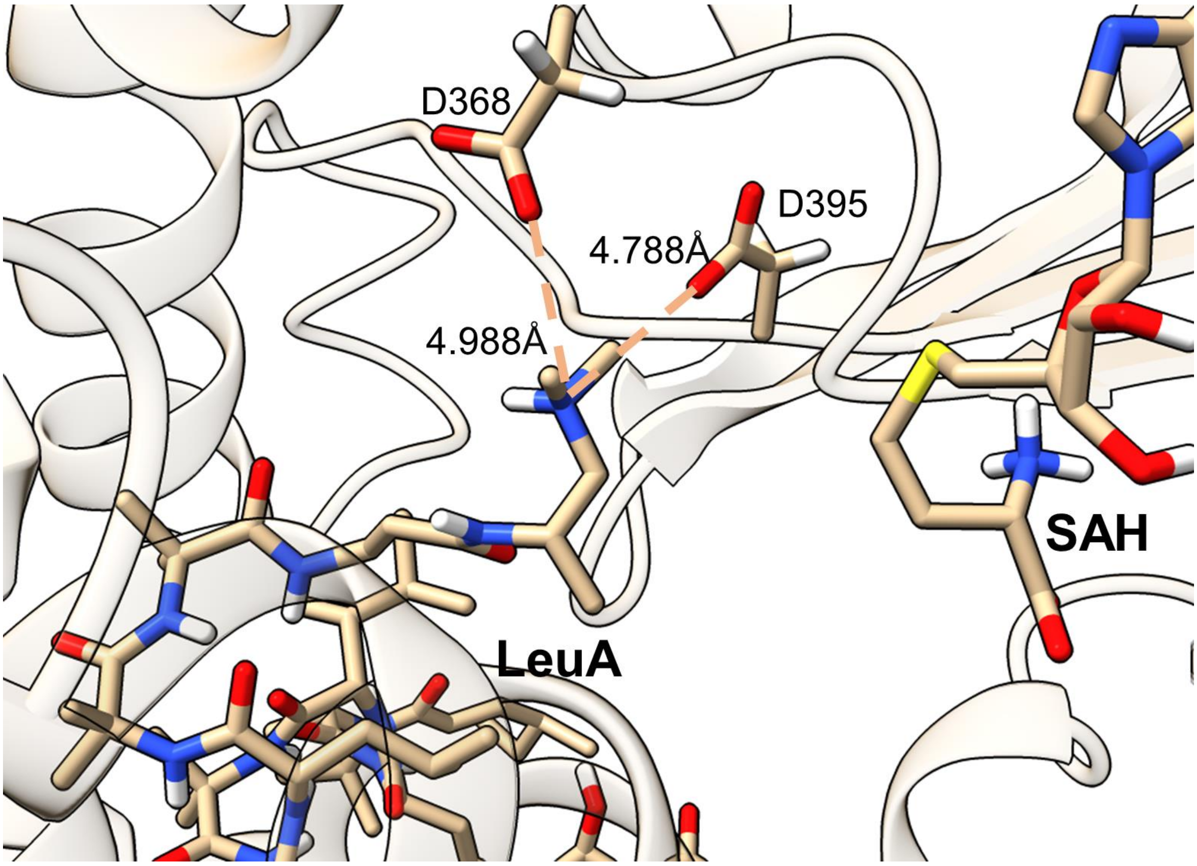

Supplementary Figure 23. Representative snapshot from molecular dynamics simulations depicting the formation of salt bridges between the ligand and D368 and D395.

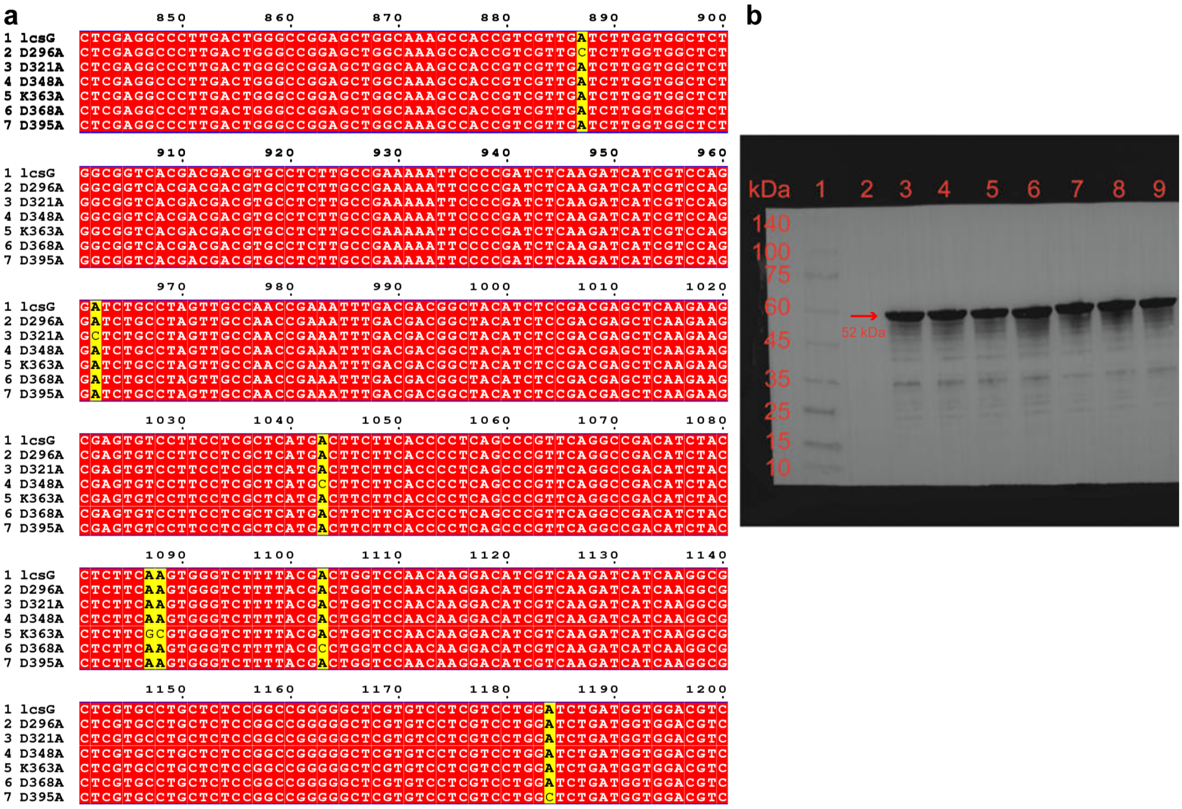

Supplementary Figure 24. a) The DNA sequencing of the plasmids of LcsG mutagenesis. White letters on a red background indicated same nucleotides. The black letter on a yellow background indicated the mutation site. **b)** Western blot analysis of LcsG mutated proteins. The expected size of proteins (52kDa) was indicated. Lane 1: Ladder; Lane 2: the negative control strain contained blunt vector; Lane 3: LcsG wild-type protein; Lane 4: LcsG-D296A; Lane 5: LcsG-D321A; Lane 6: LcsG-D348A; Lane 7: LcsG-K363A; Lane 8: LcsG-D368A; Lane 9: LcsG- D395A.

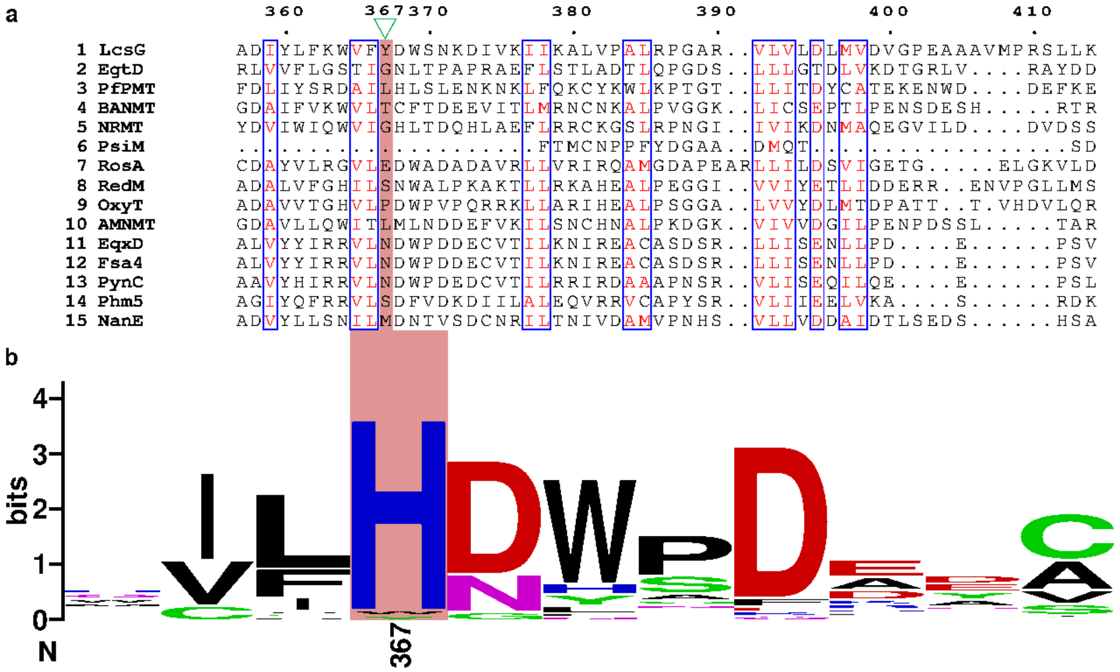

Supplementary Figure 25. Sequence comparison between the LcsG and other identified NMTs and OMTs. The residues corresponding to Y367 in LcsG were indicated with the red background and green triangle. **a)** Sequence comparison between the LcsG and NMTs. Red letters in blue boxes indicated well-conserved amino acids or similar amino acids. **b)** Generated WebLogo for the LcsG and OMTs listed in Supplementary Table 6.

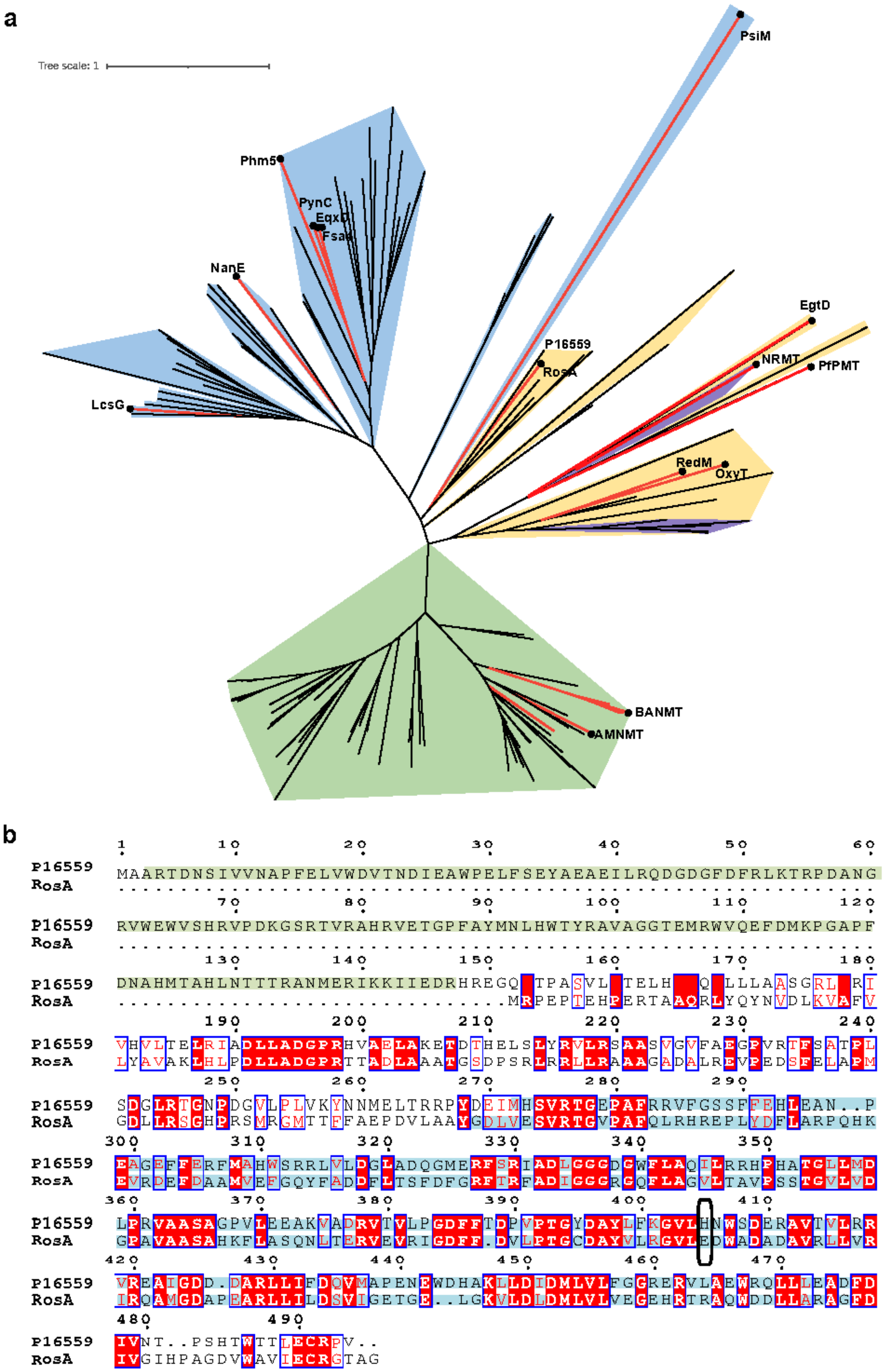

a
Tree scale: 1
PsiM
Phm5
PynC
EqxD
Fsa4
NanE
LcsG
P16559
RosA
EgtD
NRMT
PfPMT
RedM
OxyT
BANMT
AMNMT
b
1 10 20 30 40 50 60
P16559 MAARTDNSIVVNAPFELVWDVTNDIEAWPELFSEYAEAEILRQDGDGFDFRLKTRPDANG
RosA ....
70 80 90 100 110 120
P16559 RVWEWVSHRVPDKGSRTVRAHRVETGPFAYMNLHWTYRAVAGGTEMRWVQEFDMKPGAPF
RosA ....
130 140 150 160 170 180
P16559 DNAHMTAHLNTTTRANMERIKKIIEDRHREGQ[illegible]TPASVL[illegible]TELH[illegible]Q[illegible]LLLAASGRL[illegible]RI
RosA ...........................MRPEPTEHPERTAAQRLYQYNVDLKVAFV
190 200 210 220 230 240
P16559 VHVLTELRIADLLADGPRHVAELAKETDTHELSLYRVLRSAASVGVFAEGPVRTFSATPL
RosA LYAVAKLHLPDLLADGPRTTADLAAATGSDPSRLRRLLRAAAGADALREVPEDSFELAPM
250 260 270 280 290
P16559 SDGLRTGNPDGVLPLVKYNNMELTRRPYDEIMHSVRTGEPAFRRVFGSSFFEHLEAN..P
RosA GDLLRSGHPRSMRGMTTFFAEPDVLAAYGDLVESVRTGVPAFQLRHREPLYDFLARPQHK
300 310 320 330 340 350
P16559 EAGEFFERFMAHWSRRLVLDGLADQGMERFSRIADLGGGDGWFLAQILRRHPHATGLLMD
RosA EVRDEFDAAMVEFGQYFADDFLTSFDFGRFTRFADIGGGRGQFLAGVLTAVPSSTGVLVD
360 370 380 390 400 410
P16559 LPRVAASAGPVLEEAKVADRVTVLPGDFFTDPVPTGYDAYLFKGVLHNWSDERAVTVLRR
RosA GPAVAASAHKFLASQNLTERVEVRIGDFF.DVLPTGCDAYVLRGVLEDWADADAVRLLVR
420 430 440 450 460 470
P16559 VREAIGDD.DARLLIFDQVMAPENEWDHAKLLDIDMLVLFGGRERVLAEWRQLLLEADFD
RosA IRQAMGDAPEARLLILDSVIGETGE..LGKVLDLDMLVLVEGEHRTRAQWDDLLARAGFD
480 490
P16559 IVNT..PSHTWTTLECRPV..
RosA IVGIHPAGDVWAVIECRGTAG

Supplementary Figure 26. a) A phylogenetic tree showing the relationships between LcsG and other methyltransferases. The black and red lines represent OMT and NMT, respectively. Sequence origins are color-coded as follows: blue for fungi, yellow for bacteria, purple for animals and humans, green for plants, and white for protozoa. **b)** Alignment sequences of OMT (P16559) and NMT (RosA). White letters on a red background indicated strictly conserved amino acid residues. Red letters in blue boxes indicated well-conserved amino acids or similar amino acids. Additionally, the N-terminal aromatase/cyclase domain of the multifunctional protein P16559 is delineated with a green background, whereas the Methyltransf_2 domain is highlighted in blue. The black frame indicated the residue corresponding to Y367 in LcsG.

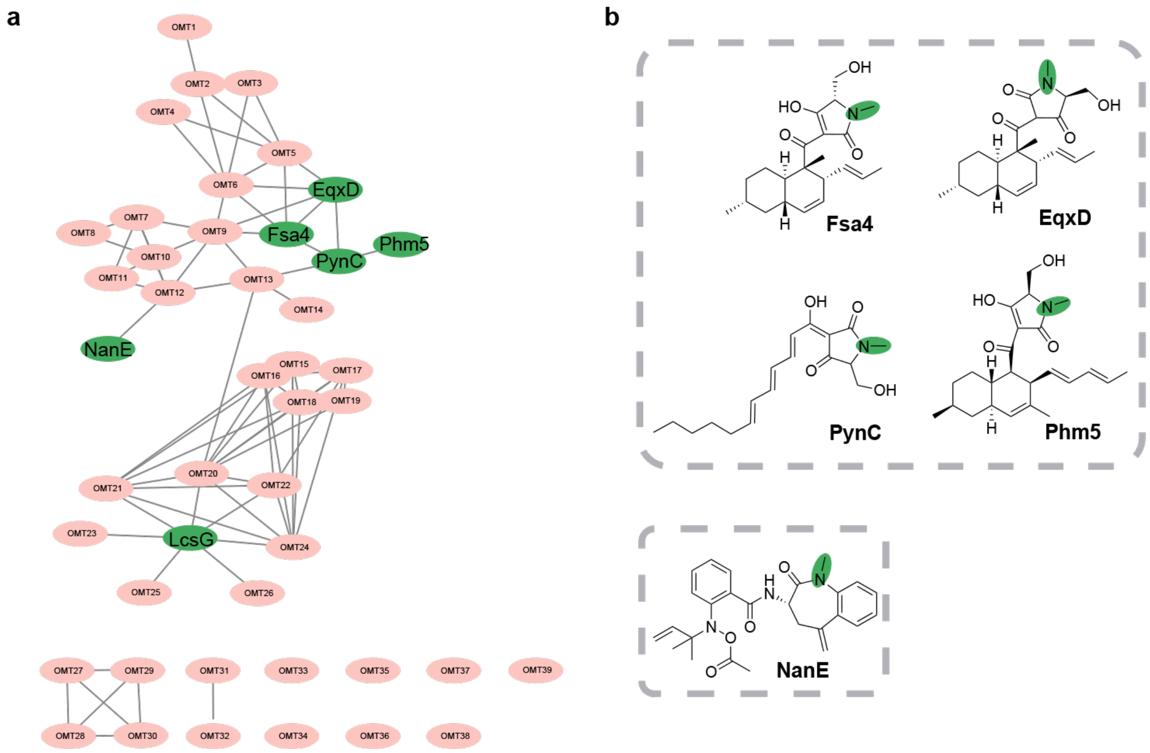

Supplementary Figure 27. a) Sequence similarity network (SSN) of LcsG and other fungi-derived methyltransferases from PF00891 (alignment score ≥10). **b)** Substrates of the NMT FsaA, PynC, EqxD, and Phm5. Catalytic sites are highlighted in green.

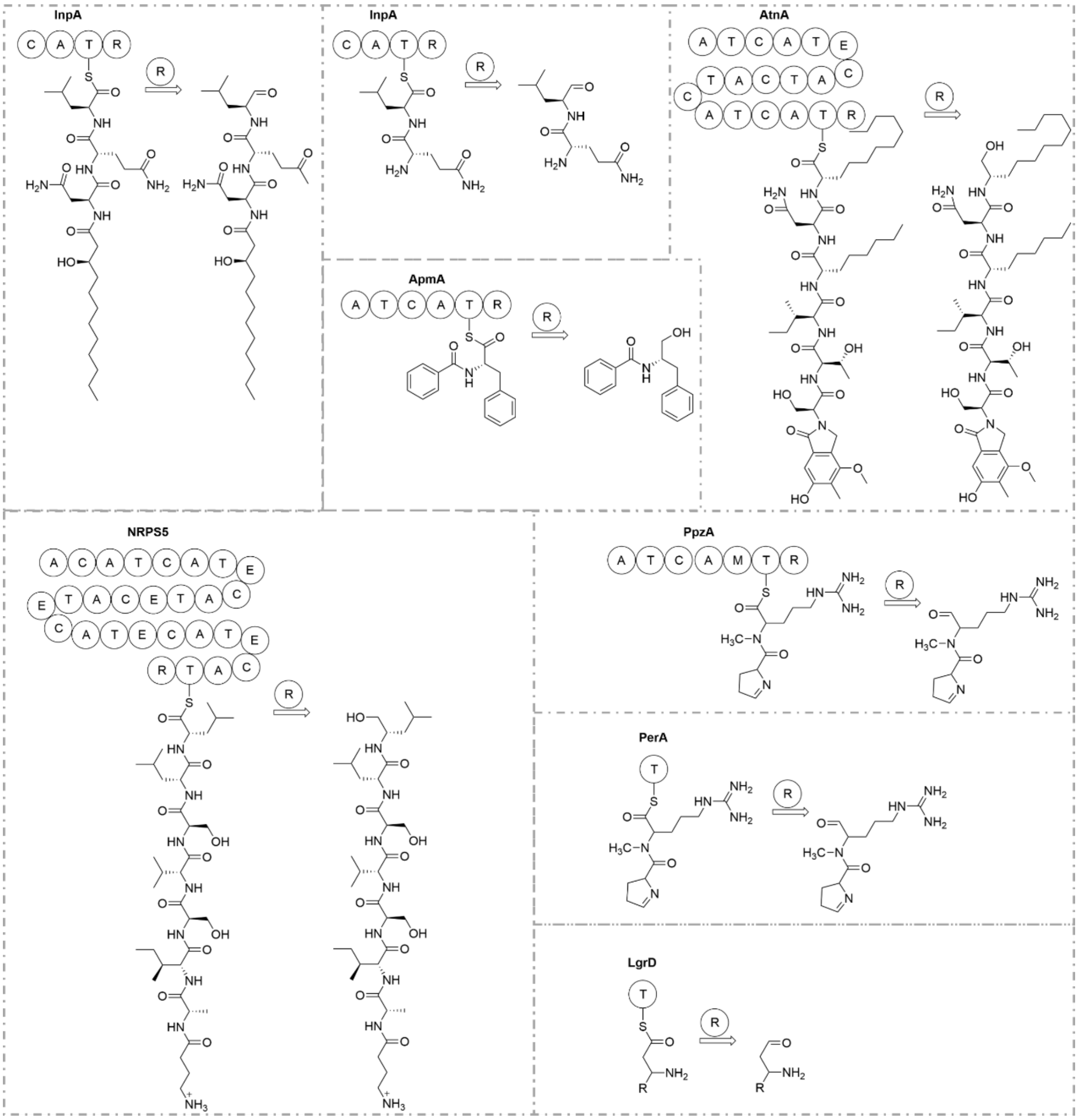

Supplementary Figure 28. The termination step mediated by the reductase (R) domain in the biosynthesis of NRPs whose gene contained similar terminal modules with that of LcsG.

Related to Figure 2a

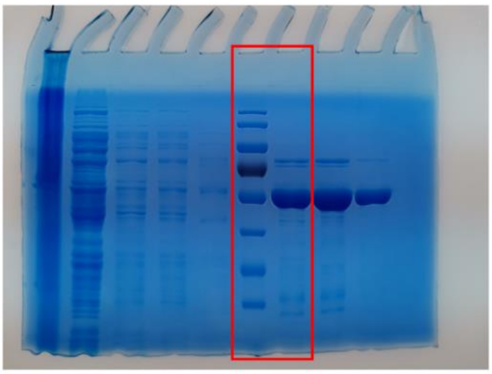

Related to Supplementary Figure 2

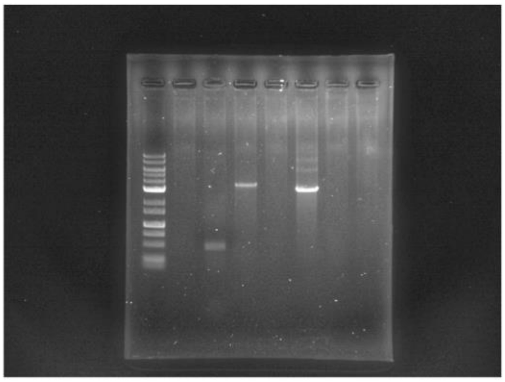

Related to Supplementary Figure 24b

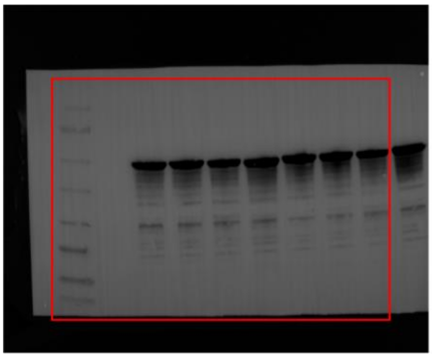

Corresponding Ponceau S Staining Image for Supplementary Figure 24b

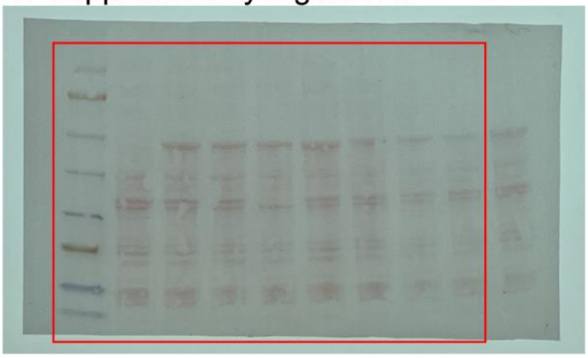

Supplementary Figure 29. Uncropped images of Fig. 2a, Supplementary Fig. 2, and Supplementary Fig. 24b.
