# Supplementary material for: Characterization of a methyltransferase for iterative N-methylation at the leucinostatin termini in Purpureocillium lilacinum
Source: Commun Biol. 2024 Jun 22;7:757. doi: 10.1038/s42003-024-06467-0 (PMC11193748; doi:10.1038/s42003-024-06467-0)
Supplement: Supplementary file 3 — Description of additional supplementary files [file 42003_2024_6467_MOESM3_ESM.pdf]

Description of Additional Supplementary Files

File name: Supplementary Data 1

Description: Information of experimentally confirmed methyltransferase analogous to LcsG used for phylogenetic tree construction in this study.

File name: Supplementary Data 2

Description: The source data behind the graphs in the paper.

File name: Supplementary Data 3

Description: All initial coordination and simulation input files of the MD simulations
