# Supplementary material for: Characterization of a methyltransferase for iterative N-methylation at the leucinostatin termini in Purpureocillium lilacinum
Source: Commun Biol. 2024 Jun 22;7:757. doi: 10.1038/s42003-024-06467-0 (PMC11193748; doi:10.1038/s42003-024-06467-0)
Supplement: Supplementary file 7 — Life sciences reporting summary [file 42003_2024_6467_MOESM7_ESM.pdf]

nature portfolio

Corresponding author(s): Yan Li

Last updated by author(s): 2024-05-23

Reporting Summary

Nature Portfolio wishes to improve the reproducibility of the work that we publish. This form provides structure for consistency and transparency in reporting. For further information on Nature Portfolio policies, see our Editorial Policies and the Editorial Policy Checklist.

Statistics

For all statistical analyses, confirm that the following items are present in the figure legend, table legend, main text, or Methods section.

n/a	Confirmed	
☐	☒	The exact sample size (*n*) for each experimental group/condition, given as a discrete number and unit of measurement
☐	☒	A statement on whether measurements were taken from distinct samples or whether the same sample was measured repeatedly
☒	☐	The statistical test(s) used AND whether they are one- or two-sided<br>*Only common tests should be described solely by name; describe more complex techniques in the Methods section.*
☒	☐	A description of all covariates tested
☒	☐	A description of any assumptions or corrections, such as tests of normality and adjustment for multiple comparisons
☐	☒	A full description of the statistical parameters including central tendency (e.g. means) or other basic estimates (e.g. regression coefficient) AND variation (e.g. standard deviation) or associated estimates of uncertainty (e.g. confidence intervals)
☒	☐	For null hypothesis testing, the test statistic (e.g. *F*, *t*, *r*) with confidence intervals, effect sizes, degrees of freedom and *P* value noted<br>*Give P values as exact values whenever suitable.*
☒	☐	For Bayesian analysis, information on the choice of priors and Markov chain Monte Carlo settings
☒	☐	For hierarchical and complex designs, identification of the appropriate level for tests and full reporting of outcomes
☒	☐	Estimates of effect sizes (e.g. Cohen's *d*, Pearson's *r*), indicating how they were calculated

Our web collection on statistics for biologists contains articles on many of the points above.

Software and code

Policy information about availability of computer code

Data collection	N. A.
Data analysis	All data were exported by GraphPad Prism 8. The sequence similarity networks was visualized using Cytoscape version 3.9.1.

For manuscripts utilizing custom algorithms or software that are central to the research but not yet described in published literature, software must be made available to editors and reviewers. We strongly encourage code deposition in a community repository (e.g. GitHub). See the Nature Portfolio guidelines for submitting code & software for further information.

Data

Policy information about availability of data

All manuscripts must include a data availability statement. This statement should provide the following information, where applicable:

- Accession codes, unique identifiers, or web links for publicly available datasets
- A description of any restrictions on data availability
- For clinical datasets or third party data, please ensure that the statement adheres to our policy

Information of experimentally confirmed methyltransferase analogous to LcsG used for phylogenetic tree construction in this study was in Supplementary Data 1. The source data behind the graphs was in Supplementary Data 2. All initial coordination and simulation input files of the MD simulations was in Supplementary Data 3.

Human research participants

Policy information about studies involving human research participants and Sex and Gender in Research.

Reporting on sex and gender	N. A.
Population characteristics	N. A.
Recruitment	N. A.
Ethics oversight	N. A.

Note that full information on the approval of the study protocol must also be provided in the manuscript.

Field-specific reporting

Please select the one below that is the best fit for your research. If you are not sure, read the appropriate sections before making your selection.

☒ Life sciences ☐ Behavioural & social sciences ☐ Ecological, evolutionary & environmental sciences

For a reference copy of the document with all sections, see nature.com/documents/nr-reporting-summary-flat.pdf

Life sciences study design

All studies must disclose on these points even when the disclosure is negative.

Sample size	No calculations were performed to determine the sample size. It is generally accepted that three biological replicates are sufficient for inferential analysis
Data exclusions	No data were excluded.
Replication	Each experimental condition was replicated in biological triplicates in every given experiment, and sufficient agreement was found across all data points in each experimental condition.
Randomization	Colonies were selected randomly from agar plates when being prepared for experimental pre-cultures, a single colony represents one biological replicate. To measure the performance of strains, three single colonies of target strain were randomly picked up as biological replicates. The sample allocation was not available because patient or human population data was not involved in this study.
Blinding	Microbial strains were used in this study.Unlike the clinical trials, blind is generally not necessary for the microbial fermentation experiments, in which a microorganisms is the object of study.

Reporting for specific materials, systems and methods

We require information from authors about some types of materials, experimental systems and methods used in many studies. Here, indicate whether each material, system or method listed is relevant to your study. If you are not sure if a list item applies to your research, read the appropriate section before selecting a response.

Materials & experimental systems

n/a	Involved in the study	
☐	☒	Antibodies
☒	☐	Eukaryotic cell lines
☒	☐	Palaeontology and archaeology
☒	☐	Animals and other organisms
☒	☐	Clinical data
☒	☐	Dual use research of concern

Methods

n/a	Involved in the study	
☒	☐	ChIP-seq
☒	☐	Flow cytometry
☒	☐	MRI-based neuroimaging

Antibodies

Antibodies used	anti-His antibody (TransGen Biotech Ltd, Beijing, China)
Validation	western blot
